# Per-Lesion Mask-Supervised Attention for Multi-Label Diabetic Retinopathy Lesion Detection

**DOI:** 10.3390/diagnostics16213662

**Published:** 2026-11-05

**Authors:** Wael Alghamdi, Fawaz Alsolami, Mostafa Saleh

**Affiliations:** 1 Department of Computer Science, Faculty of Computing and Information Technology, King Abdulaziz University, Jeddah 21589, Saudi Arabia; falsolami1@kau.edu.sa; 2 Department of Information Systems, Faculty of Computing and Information Technology, King Abdulaziz University, Jeddah 21589, Saudi Arabia; msherbini@kau.edu.sa

**Keywords:** diabetic retinopathy, microaneurysms, retinal hemorrhages, hard exudates, soft exudates, explainable AI, attention supervision, cross-attention, deep learning, fundus photography

## Abstract

**Background/Objectives:** Deep learning models for diabetic retinopathy (DR) usually report a severity grade, and their attention maps explain that grade rather than individual lesions. We aimed to detect microaneurysms, hemorrhages, hard exudates and soft exudates (cotton-wool spots) separately, each with an attention map trained to fall on that lesion. **Methods:** An EfficientNet-B4 and Transformer encoder, pretrained on severity grades, feeds four lesion queries that each return a score and an attention map; a localization loss trains each map toward its lesion mask. We trained it on DDR and TJDR, tested it on their test sets and on two external sets (IDRiD and e-ophtha), and compared it with a U-Net. **Results:** The mean areas under the curve (AUCs) were 0.93–0.94 for hemorrhages, 0.90–0.96 for hard exudates and 0.81–0.90 for soft exudates; microaneurysms reached 0.91–0.96, except on DDR (0.58). We analyze this drop in detail. Attention ranked lesion pixels above the rest of the retina with an AUC-Judd of 0.948–0.996. Without the localization loss, detection was slightly lower, and attention moved to regions outside the lesions that still raised the prediction. The model detected lesions better than U-Net (mean AUC of 0.876 against 0.853) and, unlike the latter, also learned from images that have labels but no masks: with masks on half of the training images, detection and AUC-Judd were nearly unchanged. **Conclusions:** Supervising each lesion’s attention with its own mask gives a per-lesion score and a map that falls on that lesion, without reducing detection.

## 1. Introduction

**Figure 1:**
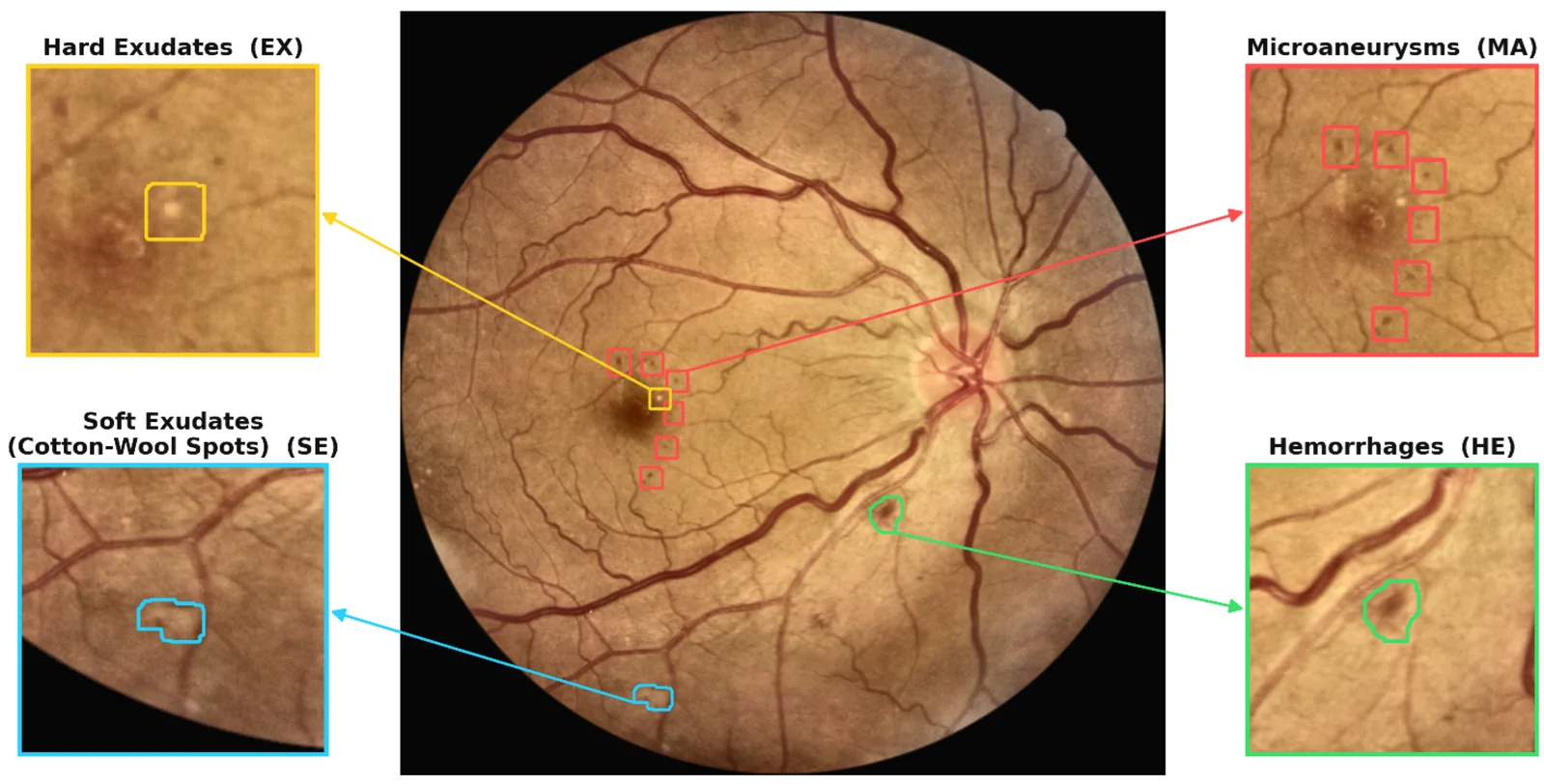
The four diabetic retinopathy lesions on a fundus image: microaneurysms (MAs), hemorrhages (HEs), hard exudates (EXs), and soft exudates (SEs).

Diabetic retinopathy (DR) is a microvascular complication of diabetes and a leading cause of vision loss among adults [1,2]. The number of adults with diabetes is projected to rise from 537 million in 2021 to 783 million by 2045 [3]. About one in three people with diabetes shows some retinopathy, and about one in ten has vision-threatening disease [4]. In 2020, an estimated 103 million adults had diabetic retinopathy, a figure projected to reach 160 million by 2045 [5]. Clinically, the disease is diagnosed through a small number of focal lesions: microaneurysms (MAs), hemorrhages (HEs), hard exudates (EXs), and soft exudates (SEs; also called cotton-wool spots), as shown in Figure 1; the type of these lesions and their extent determine the patient’s severity grade on the international scale [6,7]. A useful model must do more than just determine the severity grade; it must also indicate the lesions present and show that it examines the correct locations when it does so.

Most deep learning models on fundus images have approached this either as image-level grading or as pixel-level lesion segmentation, and large, clinically validated screening systems now exist [8,9], with reader studies showing that saliency overlays change how graders use them [10]. Public datasets with labels and masks are also available and support both directions [11,12,13,14], although those with pixel masks remain few and small (Section 2.3). Multi-label detection sits between these two approaches: a single image usually contains several lesion types together, so the problem is multi-label by nature, and the four lesions differ enough in size and appearance that treating them together under a single saliency map leads to loss of information; class imbalance across the four lesion types further complicates the learning process, and is typically addressed by re-weighting the loss [15,16]. Microaneurysms, often only a few pixels wide, raise two questions most sharply: whether the model finds them and whether we can confirm that it focuses on them rather than on something nearby.

Per-lesion attention answers both questions at once. Query-based Transformers are one practical way to build it [17,18,19,20], and lesion-aware DR designs have used lesion queries in this way [21,22]. In those two designs the queries serve a grade rather than a lesion, so no query is committed to one lesion type. Hence we enforce this separation: one query per lesion, each query focused on the location of its lesion.

Interpretability of the models is usually produced after training is complete. For example, Grad-CAM and Grad-CAM++ [23,24], Integrated Gradients [25], and Attention Rollout [26] generate the saliency map after training ends. These methods share a practical weakness when applied to fundus images, since the map is computed for one prediction at a time, not for each lesion separately, and part of its mass leaks onto the black background outside the retina. The alternative is to supervise attention during model training. GAIN [27], in its GAIN_ext_ variant, trains the attention map itself against a small set of pixel masks and improves both the localization and the map. We therefore apply this idea to the per-lesion queries in order to address the background-leakage problem that the post hoc methods expose. Papadopoulos et al. [28] reached the same point from the other side: their attention localizes DR lesions on its own, and they name the use of pixel masks to sharpen it as an open problem.

To the best of our knowledge, there is no prior lesion-detection method that combines these three elements: detecting each lesion separately, attention being supervised by the lesion masks, and a quantitative test, one that does not reuse the training masks, of whether attention drives prediction.

The main contributions of this work are as follows:

- A detection model with a separate, independent attention query for each lesion, returning a score and an attention map for every lesion in a single pass.
- A localization loss training each lesion’s attention map on its own mask, so the explanation is learned during training, not derived after it.
- An evaluation testing both whether each lesion’s attention falls on the lesion and whether the prediction depends on it.
- A per-lesion evaluation on DDR, TJDR and two datasets not used in training, with a comparison against a U-Net and an analysis of the low microaneurysm result on DDR.

Our model rests on a two-stage training structure. The shared encoder, an EfficientNet-B4 backbone [29] followed by a Transformer encoder, is first pretrained on the five-class International Clinical Diabetic Retinopathy (ICDR) severity grades. In the second stage it is fine-tuned on lesion masks, where the four lesion queries and the localization loss are introduced. This shared encoder converts the image into a grid of spatial tokens; then four lesion queries pool these tokens, one query for each of MA, HE, EX, and SE. Each query focuses only on the spatial tokens, without looking at the other queries, and returns a score and a spatial map for its own lesion.

This paper continues as follows: Section 2 reviews related work. Section 3 describes the materials and methods: datasets and labels; preprocessing; the two-stage architecture with its localization loss; the detection metrics; the evaluation of attention by AUC-Judd, by deletion and insertion, and by Attention-Inside-Retina (AIR); additional experiments; and implementation and statistics. Section 4 reports the results: grade-level pretraining, per-lesion detection on the four datasets, the analysis of the low microaneurysm result on DDR, the attention maps, the deletion and insertion tests, and the ablation of the localization loss. It then tests the effect of the loss weight λ and of each training component, compares the model with a U-Net, trains it with half of the masks, examines the attention on images that lack a lesion but contain other lesions, and tests robustness to changes in image quality. The findings are discussed in Section 5, and Section 6 concludes.

## 2. Related Work

Deep learning on fundus images is usually classified into three main tasks: image grading, lesion detection, and pixel-level lesion segmentation. The role that attention plays differs greatly across these tasks. We therefore classify diabetic retinopathy studies by task. It is the task, not the underlying architecture, that decides whether attention must name and locate a lesion or merely support a grade. Although the tasks differ, many of the underlying methods are shared. Query-based Transformers (which learn a small set of vectors that pool the image into per-target representations) extend from the object queries in DETR [17] to their multi-label classification form in Query2Label [18] to class-token designs such as MCTformer [19,20] that read localization from token-to-patch attention. Other Transformer models do not use queries at all: C-Tran [30] feeds label embeddings and their positive, negative, or unknown state into an encoder and trains by masking labels. Most of these are built on self-attention [31] and the Vision Transformer [32], usually with hierarchical or deformable backbones [33,34]. Convolutional networks [35] and retinal foundation models [36] are also still common. Since a fundus image usually holds several lesions at once, the task is multi-label, with class imbalance and co-occurring labels. This is handled at the loss level, using re-weightings such as focal loss [15] and asymmetric loss [16].

### 2.1. Image-Level DR Grading

Image-level models predict a single severity grade per image, and the strongest systems reach clinical-grade agreement on the five-point scale. The screening models by Gulshan et al. [9] and Ting et al. [8] established this. Attention-driven networks followed. CABNet [37] adds a category-attention block to counter grade imbalance. GREEN [38] re-ranks grades over a class-relation graph. Attention appears inside many of them: the lesion-aware Transformer by Sun et al. [21] and the lesion-query pooling of LQAP [22] attach lesion-oriented attention to a grading head, while SSiT [39] guides self-supervised pretraining with saliency, and MSTNet [40] steers grading with multi-instance pooling. In the attention-based designs above, attention only supports the grade; no query is tied to a single lesion, the maps mix the lesions together, and no one checks whether they drive the prediction. The authors of CABNet [37] show that their model gives a grade and a coarse location but not the lesion type, which they leave to future work. However, a screening report has to name each lesion and show where it is, and a grade on its own cannot do that.

### 2.2. Lesion Detection and Localization

Quellec et al. [41] detect lesions from referable-DR labels alone, reading them off a single-channel sensitivity map obtained by constraining the input perturbation to preserve hue and by optimizing the sparsity of that map during training, with free-response receiver operating characteristic (FROC) areas reported on DiaretDB1 for the same four lesion types studied here. Because the network carries a single referable-DR output, it produces one such map; per-lesion separation is obtained by exporting different training checkpoints, each of which happens to peak on a different lesion, rather than from the architecture itself. Within DR, weakly supervised lesion localization from image-level labels has been explored within interpretability frameworks [42]. MVCAViT [43] combines multi-view cross-attention with localization. Lesion identification has also been framed as cross-domain multi-label classification [44]. Other lesion-aware DR designs bring in category-relation attention [45], multi-task progressive learning [46], or contrastive objectives [47], but the attention that they carry is neither committed to a single lesion nor supervised toward it.

Though from another imaging domain, EL-DETR [48] pairs a deep convolutional neural network (DCNN) with separate content- and location-query attention. The separation there is content against location within a generic object query, not one query per lesion type; attention emerges from training rather than being supervised; and its maps are presented rather than scored. Where these detection methods quantify their saliency, they score whether it lands on annotated lesions, as in the FROC evaluation by González-Gonzalo et al. [42]. That measures placement, not whether the highlighted pixels are the ones the model used, and it is not decoupled per lesion. Detection and a faithful per-lesion explanation are treated as two separate problems, and the explanation is often not addressed.

Cheng et al. [49] use a graph convolutional network over label co-occurrence to classify eight lesion types at the image level, on a self-collected set of 7459 images that the authors state cannot be released. Their label set spans DR lesions alongside findings such as laser scars and cup-to-disc ratio, and microaneurysms score the lowest of the eight, which the authors attribute to the difficulty of separating small red points from the retinal background. The model produces no localization output. Jiang et al. [50] attach post hoc Guided-Grad-CAM to a multi-label classifier, thresholding the result into lesion contours, but the maps are read off a trained classifier rather than supervised toward the lesions. A multiple-instance approach by Papadopoulos et al. [28] localizes DR lesions from unsupervised attention as a by-product of referable-DR detection but explicitly leaves the use of pixel-level lesion masks to sharpen that localization as an open problem.

Two recent systems sit closer to the setting studied here. DeepDR [51] builds a ResNet lesion-detection module and a Mask R-CNN segmentation module over a shared feature extractor, covering the same four lesion types studied here. Its target is DR screening, and the lesion features are concatenated into the grading sub-network to improve grading. Its unit of evaluation is the quadrant, since the lesion-detection module classifies whether a quadrant of the retinal image contains a given lesion, and it was developed on a private cohort whose splits are not released.

CLAT [52] represents each lesion as a learnable token in a Transformer encoder and reaches a diagnosis through cross-attention between disease tokens and lesion tokens, supervising the lesion tokens with image-level labels that the authors derive from the existence of a mask. Its target is retinal disease diagnosis, where the lesion concepts carry the explanation of the disease decision. Its lesion detection is reported as the area under the receiver operating characteristic (ROC) curve (AUC), averaged over the four lesions with no per-lesion breakdown, on a custom 1500-image DDR subset under 10-fold cross-validation rather than on the official DDR lesion test split.

Across these methods, attention serves the model’s own target, whether a grade, a referral decision, or a disease label. We treat the per-lesion map as an output in its own right: supervised toward its lesion and scored independently.

### 2.3. Pixel-Level Lesion Segmentation

A third group of methods segments the four lesions at the pixel level. Early methods such as L-Seg [53] established how hard this task is. Recent methods improve accuracy with multi-scale, Transformer, relation-based, deformable-convolution, feature-reassembly, and state-space (Mamba-style) designs [54,55,56,57,58,59,60], with some building on DeepLab backbones [61] and some fine-tuning a foundation model such as SAM [62], so far for one lesion at a time, trained on dense mask datasets including DDR, IDRiD, and FGADR [63]. The difficulty is strongly lesion-specific: RTNet [54], for instance, reports pixel-level areas under the precision–recall curve (AUPRs) of 0.57 for hard exudates and 0.12 for microaneurysms on DDR. On the easier IDRiD set, the same two lesions reach 0.90 and 0.49. Later methods roughly double the DDR figure, and the strongest reported AUPR for microaneurysms on that test set is near 0.28 [58]. The gap reflects how few pixels the smallest lesions occupy. Segmentation localizes lesions by design but at a cost that matters for screening: it needs pixel-level labels at training, and it produces a mask. It does not give a per-lesion score for whether a lesion is present. Public datasets with such masks are also few and small, a shortage noted by Zhou et al. [63]: IDRiD provides masks for 81 images, TJDR for 561, DDR for 757 and FGADR for 1842, against 15,429 graded images in DDR and APTOS alone. Some segmentation networks, such as RTNet [54] and MSGDA-Net [55], also couple lesions to vessels or to one another, which helps the pixel task but works against the property we want: a query whose attention answers for a single lesion. Because these networks output masks rather than a per-lesion score, their reported results are pixel-level and cannot be compared with our image-level detection. Instead, Section 4.7 compares our model with a U-Net that we trained under the same conditions, so that both can be scored at the image level and pixel level.

### 2.4. Attention: Explanation and Its Supervision

Across image grading, lesion detection, and lesion segmentation, the key question is how a model’s attention is produced and judged. Explanations are most often computed after training: class activation mapping [64] and its gradient-weighted successors Grad-CAM and Grad-CAM++ [23,24], Integrated Gradients [25], and for Transformers, Attention Rollout [26] and relevance-propagation methods [65]. These attributions are tied to particular architectures: the Grad-CAM family to convolutional classification heads, and Attention Rollout to stacks of self-attention. Neither maps cleanly onto a decoupled per-lesion cross-attention pooling design. Our attention, instead, is built into the model and produced per lesion in the forward pass, whereas any post hoc map is computed afterward on top of a trained network. We later compare native attention with Integrated Gradients, which returns a separate map for each lesion query. Attention Rollout also applies to the encoder, but it aggregates the shared self-attention stack into one map, so it cannot be read per lesion. On fundus images, these post hoc maps share a concrete failure: they are computed one prediction at a time, not per lesion, and a non-trivial share of their mass lands on the black border outside the retina (Section 4.4). When such maps are scored against expert lesion annotations on IDRiD, agreement is low. A systematic comparison of ten heatmapping techniques reported consistency scores between only 0.21 and 0.51, even for the best combination [66]. Whether raw attention is an explanation at all has been debated [67], and saliency maps do not always pass basic sanity checks [68], which is why qualitative inspection alone is not enough [69,70]. The alternative supervises attention during training: GAIN [27], in its GAIN_ext_ variant, adds a squared-error term between the attention map and a small set of pixel masks and improves localization and the map together. That supervision, though, acts on a gradient-derived map and serves segmentation, and it is scored against masks of the same kind it was trained on. To our knowledge, no prior work applies this supervision to per-lesion DR queries or tests, for such queries, whether attention drives prediction. Three requirements are still not met in combination: per-lesion detection paired with an attention map decoupled per lesion, attention supervised toward the lesion rather than left to emerge, and an evaluation that tests whether attention drives prediction without reusing the training masks. Table 1 summarizes the representative methods by task, mechanism, explanation, datasets, and key limitation. Our method is built to satisfy such requirements.

## 3. Materials and Methods

### 3.1. Datasets and Labels

We use four public datasets: DDR [11] and TJDR [14] provide the pixel-level lesion masks used for in-domain fine-tuning and testing; IDRiD [12] and e-ophtha [13] are kept out of training entirely and act as out-of-domain generalization tests, collected with different cameras and from different populations. Grade-level pretraining additionally uses the DDR and APTOS 2019 Blindness Detection [71] severity labels, which are available for many more images than the masks. Table 2 summarizes each source with its role, image count, and mask format. *Stage 1 data.* The shared encoder is first pretrained on image-level severity labels from DDR and APTOS. Since this stage only initializes the backbone and no grade-level test metric is reported, every grade-labeled image outside the validation set is used for training: the DDR grading training and test splits (5833 and 3574 images) and the APTOS training and test splits (2930 and 366) are merged into a single 12,703-image training set, while the DDR grading validation split (2360) and the APTOS validation split (366) form a 2726-image validation set. DDR’s ungradable grade-5 images are excluded, so only ICDR grades 0–4 are used. The grade balance is kept across the two sets (per-grade differences below one percentage point), and pretraining performance is reported on the validation set only.

*Stage 2 data.* The pixel-annotated images are then split again for the detection task. The DDR lesion training and validation splits (383 and 149 images) and the TJDR training split (448) are pooled into a single set of 980 images and divided in a 85/15 ratio into 832 training and 148 validation images. The split is stratified jointly by source dataset and by per-lesion presence, so the DDR/TJDR proportion (about 54/46) and the positive rate of every lesion (MA, HE, EX, and SE) are matched across the two sets to within one percentage point. The official DDR (225) and TJDR (113) lesion test splits are kept separate for in-domain evaluation, and IDRiD (all 81 pixel-annotated images) and e-ophtha (148/233 MA and 47/35 EX positive/negative images) are kept out of training entirely as out-of-domain generalization tests.

Some lesions are much more common than others, and this imbalance affects both training and how we read the numbers. Table 3 reports, for each test set, how many images contain each lesion (positive) and how many do not (negative). Soft exudates are the rarest in the training set, which is why we later read average precision together with AUC and treat SE as the most imbalanced lesion.

### 3.2. Preprocessing

**Figure 2:**
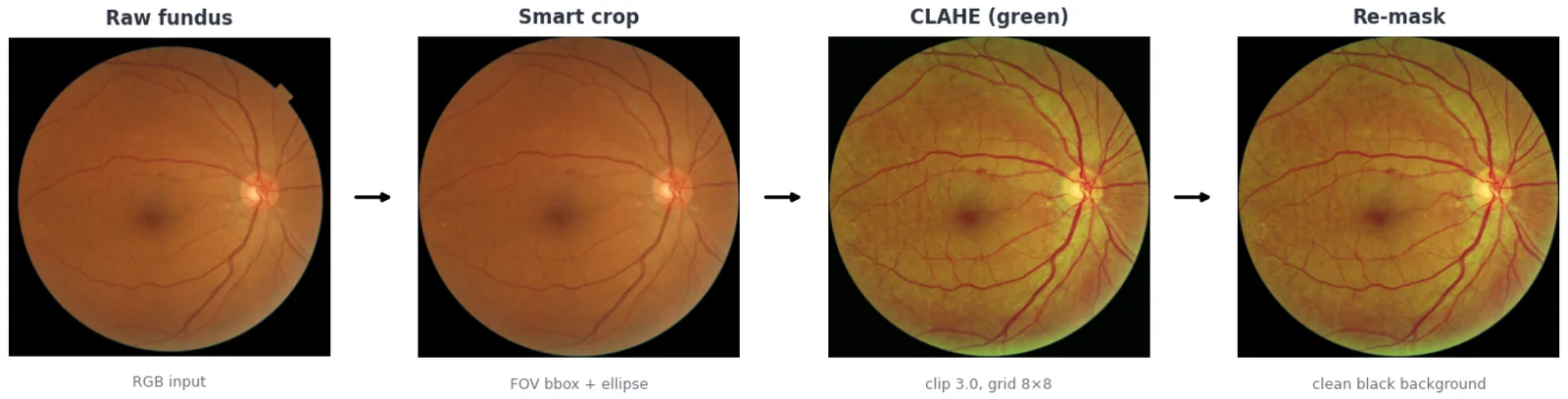
Preprocessing pipeline: crop, contrast-limited adaptive histogram equalization (CLAHE), and re-mask.

Fundus images come at different resolutions and with the retinal disc placed differently within a frame (Figure 2). Cropping is done from a detected field-of-view mask instead of a fixed circle. A naive center crop fails on several of our sources; IDRiD images, for instance, are cut off at the top and bottom by the camera aperture. We therefore detect the retina adaptively (thresholding brightness on a black background or a red-channel Otsu split when the background is gray), take the bounding box of that mask, and fit an inscribed ellipse. This removes only background and never clips retinal tissue, which matters because a lesion near the disc margin would otherwise be lost.

Contrast is enhanced with contrast-limited adaptive histogram equalization (CLAHE) [72] applied to the green channel (clip limit of 3.0; 8 × 8 tiles). Green carries most of the vascular and lesion contrast in color fundus photography, and limiting the enhancement to that channel sharpens the small dark lesions (microaneurysms and hemorrhages) that are otherwise hard to separate from the background. The same green-channel CLAHE step has been used before for microaneurysm detection on fundus images [73]. CLAHE also lifts the previously black corners outside the field of view to small non-zero values. We therefore re-apply the field-of-view mask after CLAHE, zeroing those corners again. If the corners were left raised, the model would pick up a spurious signal from the border, and AIR (Section 3.6.3) would be measuring contrast artifacts instead of where the model looks. Images are then resized to 1024 × 1024 and normalized with ImageNet statistics.

We keep the augmentation simple and mostly geometric, built with the Albumentations library [74]: horizontal and vertical flips, small rotations of up to 15°, and a shrink-only affine transform (scale between 0.95 and 1.0, with small translation and shear). This transform pads instead of cropping, so a peripheral lesion is never pushed out of the frame. We narrowed this scale range after checking that stronger shrinkage (scale of 0.85) reduced the average microaneurysm from 4.59 to 3.99 pixels at 1024 resolution, a 13% size loss on the hardest class for little added augmentation value. We do not apply photometric color jitter, elastic warping, blur, added noise, or cutout. Each of these can distort, bury, or erase a microaneurysm (a lesion only a few pixels wide), and since MA is already the hardest lesion to detect, we keep every one of its pixels intact, even though these augmentations would usually add robustness.

### 3.3. Model Architecture

The model has two parts: reading diabetic lesions and pointing, per lesion, to where it is. A shared encoder turns the image into spatial tokens, and four lesion-specific queries read those tokens into one detection score and one attention map per lesion.

**Figure 3:**
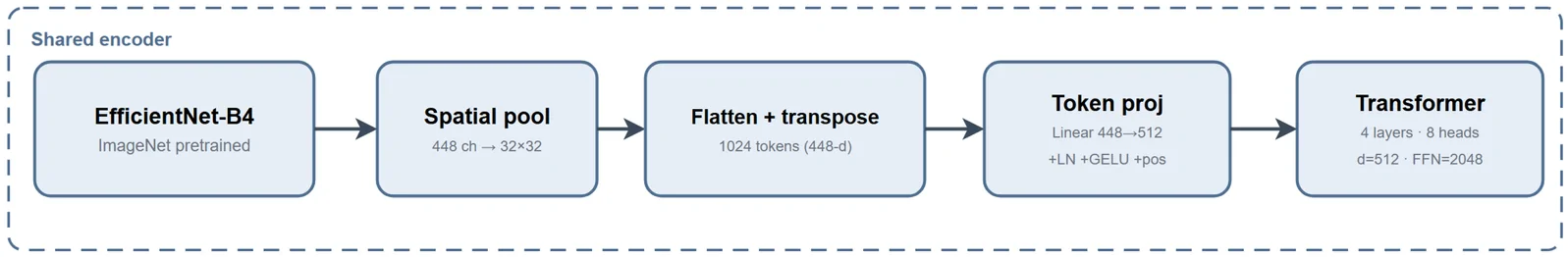
Shared encoder: an EfficientNet-B4 backbone produces 32 × 32 spatial tokens that a four-layer Transformer encoder refines into the shared representation used by both training stages.

The shared encoder turns a fundus image into a sequence of spatial tokens (Figure 3). An EfficientNet-B4 backbone, pretrained on ImageNet, reduces the image to a 448-channel feature map, which is adaptively pooled to a fixed 32 × 32 grid and flattened into 1024 spatial tokens, so that images of any input size reach the rest of the network as one sequence of the same length. A linear projection lifts each token from 448 to 512 dimensions, and a LayerNorm, a GELU non-linearity, and a learned positional embedding prepare the sequence for attention; the positional embedding is what lets the model keep track of where each token sits on the retina.

The token sequence then passes through a four-layer pre-norm Transformer encoder (512-dimensional, 8 heads, and feed-forward width of 2048), in which every token attends to every other. This is where the model forms its global read of the retina: a hemorrhage is related to the vessels around it and an exudate to the macula that it threatens, across the whole field rather than within a single patch. The output of the shared encoder is 1024 context-aware tokens, one per grid cell. These five components (backbone, spatial pool, token projection, positional embedding, and Transformer) make up the shared encoder that both stages use.

On top of the encoder sits the lesion-specific stage. Rather than collapse the encoder’s tokens into a single vector, the model keeps four learned queries, one per lesion (MA, HE, EX, and SE). This is an object-query design in the style of DETR [17], but with each query tied to a single lesion type, not a generic object slot. Each query cross-attends to the 1024 image tokens and, by design, to nothing else: the queries never attend to one another. Cross-attention uses eight heads, and their maps are averaged into the one map per lesion that the localization loss supervises and the figures show. Because the queries are decoupled, the pooling returns a separate 32 × 32 attention map per lesion marking which parts of the retina that query drew from. Each query returns a pooled 512-dimensional vector, and a small head of its own (Linear 512 → 256, GELU, dropout of 0.4, and Linear 256 → 1) turns it into that lesion’s detection score; the four heads share no weights. The same 0.4 dropout is also used in the Transformer encoder and in cross-attention. These maps are the attention weights that the model itself uses to reach its decisions, not a separate explanation computed afterward.

### 3.4. Two-Stage Training

**Figure 4:**
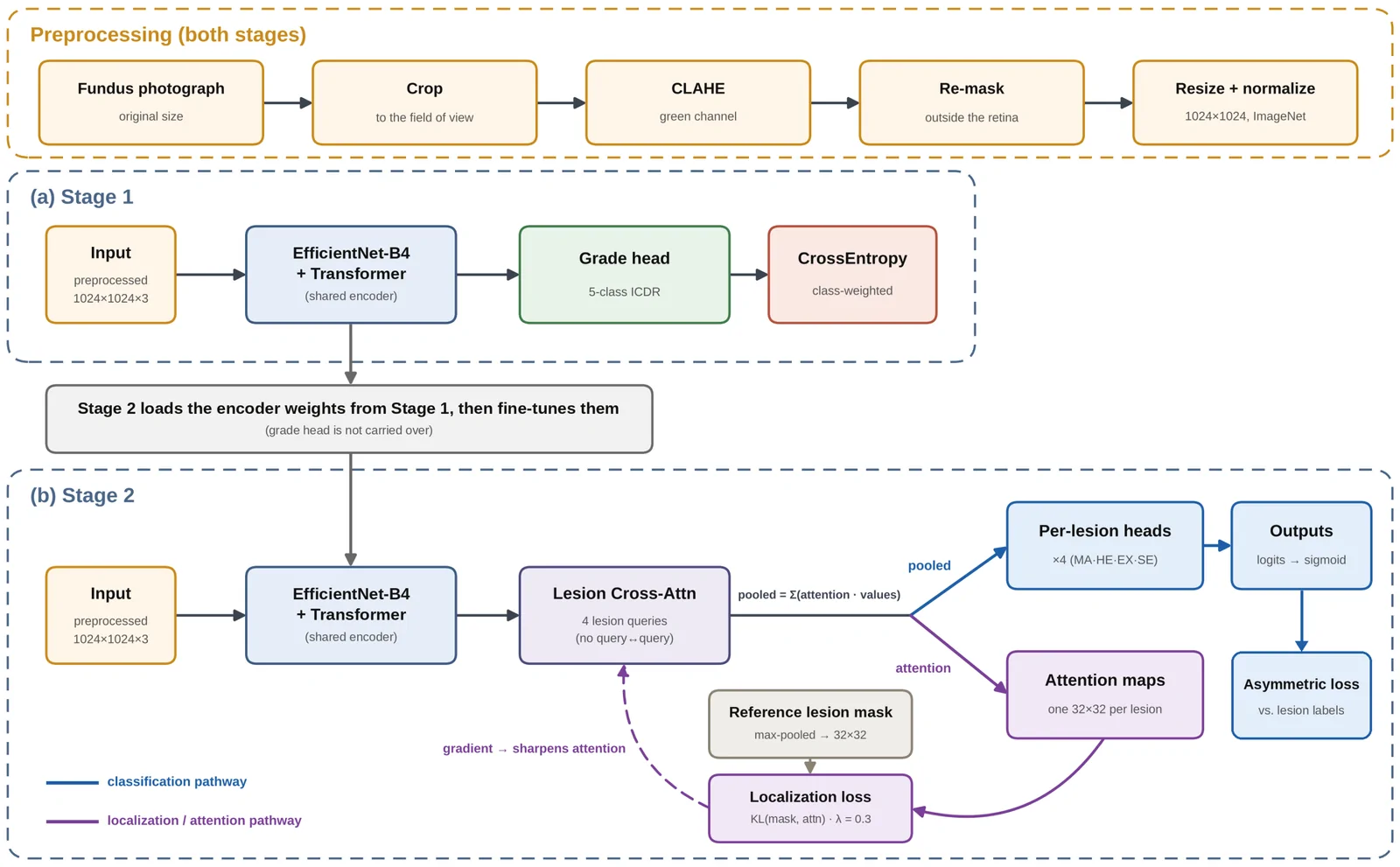
Two-stage architecture. Orange: preprocessing, applied before both stages (Section 3.2). (**a**) Stage 1: grade pretraining of shared encoder (EfficientNet-B4 + Transformer). Between stages, Stage 2 encoder is initialized with Stage 1 weights. (**b**) Stage 2: lesion fine-tuning; asymmetric loss (ASL) and localization loss are summed into one training loss (Equation (3)), whose gradient updates whole model, including encoder. Blue: classification pathway; purple: localization (attention) pathway.

The model is trained in two stages that share the encoder just described (Figure 4). Stage 1 pretrains the shared encoder on diabetic retinopathy grade, which is annotated for far more images than lesion masks are. Stage 2 loads the pretrained encoder, adds the per-lesion queries and the localization loss, and fine-tunes the whole model on the images with lesion masks. Sharing the encoder lets the lesion stage begin from a representation that already captures the general appearance of a diabetic retina instead of learning it from the small mask datasets.

#### 3.4.1. Stage 1: Grade-Level Pretraining

Before the model ever sees a lesion mask, the shared encoder is first pretrained to predict the five-class ICDR grade, an image-level label that is less detailed than a mask but available for many more images. Grade labels exist for thousands of images where pixel-level masks do not, and grading forces the backbone and Transformer to learn the general appearance of a diabetic retina (vessel changes, exudative regions, overall number and extent of lesions). Specifically, the EfficientNet-B4 backbone, spatial pooling, token projection, positional embedding, and the four-layer Transformer are trained end to end with a mean-pooled grade head, using DDR and APTOS grade labels and a class-weighted cross-entropy loss to counter the strong grade imbalance. This stage runs for 12 epochs at a batch size of 16 with a cosine-annealed learning rate. The grade head is discarded once pretraining is done; only the five shared modules carry forward. Stage 1 is evaluated on its validation set with accuracy, the share of images given the correct grade; quadratic weighted kappa (QWK), which weights each disagreement by its distance on the grade scale; and the AUC and average precision (AP) averaged over the five grades.

#### 3.4.2. Stage 2: Lesion Fine-Tuning and Localization Loss

The model is trained with two losses: a classification loss that decides which lesions are present, and a localization loss that shapes where each query attends. We describe each loss and then show how they are combined into a single objective.

The classification loss is the class-averaged asymmetric loss (ASL) [16], a multi-label variant of focal loss [15], designed for the strong class imbalance across the four lesions:

(1) $$L_{ASL}=-\frac{1}{C}\sum_{c}\left[\;y_{c}\;{\left(1-p_{c}\right)}^{\gamma_{+}}\ln p_{c}+\left(1-y_{c}\right)\;{\hat{p}}_{c}^{\gamma_{-}}\ln\left(1-{\hat{p}}_{c}\right)\;\right]$$

where C=4 is the number of lesion classes, *c* indexes them, $y_{c}\in \left\{0,1\right\}$ is the ground-truth presence label, $p_{c}$ is the predicted probability for lesion *c*, and $\gamma_{+}$ and $\gamma_{-}$ are the positive and negative focusing parameters. Setting $\gamma_{-}$ larger than $\gamma_{+}$ down-weights the many easy negative labels so that the rarer positive lesions dominate the gradient. For the negative labels, the loss does not use the raw probability $p_{c}$ directly but a shifted probability ${\hat{p}}_{c}$: the predicted probability reduced by a small margin ϵ (the ASL clip, set to ϵ=0.05) and then kept at zero or above,

$${\hat{p}}_{c}=\max\left(p_{c}-\epsilon ,\;0\right),$$

where subtracting ϵ discards easy negatives that the model is already confident about. Training then concentrates on the harder, more informative negatives.

The localization loss supervises each per-lesion attention map against its ground-truth mask (adaptive max-pooled to the 32×32 grid, which preserves microaneurysm pixels) with a Kullback–Leibler (KL) divergence term, applied only to lesions that are both labeled present and have mask pixels. Its gradient flows back through cross-attention into the encoder, sharpening each query onto its lesion:

(2) $$L_{loc}=\frac{1}{\left|P\right|}\sum_{l\in P}\sum_{i}{\tilde{m}}_{l,i}\;\ln\frac{{\tilde{m}}_{l,i}}{a_{l,i}}$$

where *P* is the set of lesions that are both present and have mask pixels, *l* indexes a lesion in that set, *i* indexes one of the 32×32 spatial tokens, $a_{l,i}$ is the attention weight that query *l* places on token *i* (so $\sum_{i}a_{l,i}=1$), and ${\tilde{m}}_{l,i}$ is the ground-truth mask of lesion *l* normalized into a distribution over the same tokens, ${\tilde{m}}_{l,i}=m_{l,i}/\sum_{j}m_{l,j}$, with $m_{l,i}$ the raw (max-pooled) mask value at token *i* and the sum running over all 32×32 tokens, so that $\sum_{i}{\tilde{m}}_{l,i}=1$. Minimizing this term pulls each query’s attention toward the true location of its lesion.

The two losses combine into a single training objective (Equation (3)):

(3) $$L=L_{ASL}+\lambda \;L_{loc},\;\lambda =0.3$$

where $L_{ASL}$ is the classification loss of Equation (1), $L_{loc}$ is the localization loss of Equation (2), and λ is the weight that balances them, fixed at 0.3 so that the mask supervision guides attention without overwhelming the detection objective. This value was selected by validation mean average precision (mAP), and the sensitivity to λ is analyzed in Section 4.6.2.

Stage 2 fine-tunes the full model (shared encoder, lesion queries, heads, and localization loss) end to end. Optimization uses AdamW [75] (learning rate of 5 × 10^−5^, backbone at 5 × 10^−6^, and weight decay of 5 × 10^−4^) with a 5-epoch gradual warm-up [76] followed by cosine decay [77], a batch size of 8 at 1024 × 1024 input, and gradient clipping at 1.0. Fine-tuning ran for up to 40 epochs with early stopping (patience of 5 on validation mean average precision (mAP)), and the reported model is the best-validation checkpoint. The asymmetric loss uses $\gamma_{-}=4$ and $\gamma_{+}=1$, with a label smoothing [78,79] of 0.1. The positive term is also weighted per lesion. The weight is the square root of the negative-to-positive image ratio in the training set, capped at 3.0, and it comes out at 0.825 for MA, 0.701 for HE, 0.850 for EX, and 1.346 for SE. MixUp [80] (α = 0.2) is applied to each batch with probability 0.5 after warm-up, with the localization loss skipped on mixed batches (masks do not mix). An exponential moving average of the weights [81] (decay 0.999) is maintained and used for all evaluation.

### 3.5. Detection Metrics

Detection is scored per lesion with the area under the ROC curve (AUC) and the area under the precision–recall curve (AP, i.e., average precision), together with F1; we take the mean average precision (mAP) across the four lesions as the primary metric, following common practice for imbalanced multi-label detection, since average precision stays informative when positives are rare. F1 is computed at per-lesion thresholds chosen to maximize F1 on the fine-tuning validation set, by a sweep from 0.1 to 0.9 in steps of 0.01, separately for each training run. The same thresholds are applied unchanged to every test set, so no test labels are used to set them. We do not report accuracy as a lesion metric: with soft exudates so heavily outnumbered by negatives, high accuracy can be reached without detecting the lesion at all. On IDRiD, every image contains MAs and EXs, and all but one contain HEs, so the AUC is undefined or not informative for these three lesions, and only average precision is reported for them; e-ophtha, which contains healthy negatives, is the more informative out-of-domain test.

To test whether microaneurysm detection depends on the images that it is compared against, we also score it against each group of MA-negative images separately: in TJDR, images with no lesions, with exudates only, and with hemorrhages; in DDR, images with hemorrhages; and the healthy images of e-ophtha. On the MA-negative images, we count small hemorrhages (at most 60 pixels, the 90th percentile of microaneurysm size), compare their diameter and green-channel contrast with those of microaneurysms, measure the share of microaneurysm attention on hemorrhages, and rank images by the number of red components (microaneurysms and hemorrhages) no larger than the 95th percentile of microaneurysm size in each set’s reference masks.

### 3.6. Evaluating the Explanation

We evaluate attention as an explanation in three ways: whether it lands on the lesion, whether it actually drives the prediction, and whether it stays on the retina. For a visual comparison, we also compute Integrated Gradients [25] for each lesion’s score; as a post hoc method, it applies to the per-lesion queries without changing the model.

#### 3.6.1. Attention Localization (AUC-Judd)

The first question is whether attention falls on the lesion. We measure this with AUC-Judd [82,83], a location-based metric from the saliency literature that treats the attention map as a pixel classifier. For each image and each lesion present in it, the map is thresholded at every distinct attention value; at each threshold, the true positive rate is the fraction of annotated lesion pixels above it, and the false positive rate is the fraction of remaining pixels above it. The score is the area under the resulting ROC curve, and chance is 0.5. Negatives are restricted to the retinal field of view, so the black border outside the retina cannot inflate the score. We report the median over images on the DDR and TJDR test sets.

The metric suits this setting for two reasons. It uses the whole attention map rather than a single location, which matters because a positive image carries many lesions at once: a DDR image positive for microaneurysms contains a median of 8.5 connected components. And because it compares lesion against non-lesion pixels pairwise, multiplying or enlarging the lesions scales both sides of every comparison and leaves the value unchanged. Scores are therefore comparable across lesions whose areas differ by an order of magnitude, and across datasets whose lesion burden differs. Overlap metrics such as intersection over union (IoU) and Dice would not be appropriate here: they would favor the localization loss, which is trained to match masks of the same kind, and penalize a coarse 32×32 map for not being pixel-level. The AUC is invariant to monotonic transformations of the map, so it reports where attention ranks the highest. Where boundary precision matters, we also report the pixel-level average precision (pixel AP) of the map against the lesion mask inside the retina, as a median over images. It rewards maps that both lie on the lesion and outline it pixel by pixel, so it is low by construction for a 32×32 map even when the map lies on the lesion; placement itself is measured by AUC-Judd.

#### 3.6.2. Faithfulness: Deletion and Insertion

An attention map can look right without driving the prediction, so we test faithfulness with the mask-free deletion and insertion metrics of RISE [84]. Over twenty steps, the highest-attention pixels are progressively replaced by the mean color of the retina and the lesion probability is tracked as it falls (deletion). Insertion is a separate test: starting from a strongly blurred copy of the image (Gaussian, σ=50 pixels), the original pixels are added back in the same order, and the probability is tracked as it rises. Both are computed on every test image that contains the lesion. We also measure where the first 10% and 20% of restored pixels fall: the share of the lesion area that they cover, and the share of them outside the retina. Faithful attention collapses the score quickly when deleted (low deletion AUC) and recovers it fast when inserted (high insertion AUC). Because each model starts from its own probability, we also report the deletion drop, i.e., the probability on the original image minus the deletion AUC, and the insertion gain, i.e., the insertion AUC minus the probability on the blurred image.

#### 3.6.3. Attention-Inside-Retina (AIR)

We quantify how well attention stays on the retina with Attention-Inside-Retina (AIR): the fraction of a query’s attention mass that falls inside the retina, not the black border outside it (Equation (4)). This is the energy-based form of the pointing game [85], with the retina in place of a lesion bounding box; the same idea has been used on chest radiographs with a lung mask [86]. A value near 1 means that almost all the attention stays within the retina. Since AIR uses the retina mask, not the lesion masks, it is defined for every lesion, including pixel-sized microaneurysms, and it is not circular with the localization objective.

(4) $$\mathrm{AIR}=\frac{\sum_{i}a_{i}r_{i}}{\sum_{i}a_{i}}$$

where *i* indexes the spatial tokens, $a_{i}$ is the attention weight at token *i*, and $r_{i}$ is the retina mask ($r_{i}=1$ inside the retina and 0 outside), so AIR∈[0,1].

### 3.7. Additional Experiments

#### 3.7.1. Ablation and Sensitivity Analyses

To see what each part of the training adds, we retrain the model without the localization loss (λ=0) and compare it with the λ=0.3 model on the same test sets. We also train it with λ=0.05, 0.1, 0.5 and 1.0 and remove each of the other training components in turn: Stage 1 pretraining, the asymmetric loss (replaced by binary cross-entropy (BCE) with and without label smoothing (LS)), MixUp, and the moving average of the weights (EMA), keeping everything else unchanged.

#### 3.7.2. Comparison with a Segmentation Baseline and Training with Half of the Masks

To compare with segmentation under the same conditions, we train a U-Net with the same EfficientNet-B4 encoder, initialized from the same Stage 1 weights and trained on the same images and masks with the same schedule and early stopping. Its decoder upsamples the encoder features in four stages with skip connections and outputs one probability map per lesion at 512×512, half the input size. It is trained with binary cross-entropy plus Dice loss, the usual losses for lesion segmentation; because the decoder starts from random weights while the encoder is pretrained, its learning rate is ten times that of the encoder. To turn a map into an image-level score, we take its maximum, so a lesion counts as present when any part of its map is high. Because mask supervision relies on fewer than 1000 images, we also test how much the model depends on their number: we remove the masks from a random half of the training images (416 of 832) and train the model on all 832 images, with masks on half of them and image-level labels alone on the other half.

#### 3.7.3. Attention on Images with Confounding Lesions

The model produces an attention map for every lesion in every image, even when the lesion is absent. For each target lesion, we take the test images that do not contain the target lesion but contain other lesions. On these images, we measure how much of the target lesion’s attention map lies on the other lesions and how well the model separates them from the images that contain the target lesion.

#### 3.7.4. Robustness to Image Degradation

We evaluate one of the λ=0.3 models on altered copies of the DDR and TJDR test images: darker (×0.7), brighter (×1.3), lower contrast (×0.7), blurred (σ=2), saved with JPEG compression at quality 30, and with added sensor noise (σ=8 gray levels), each applied to the photograph before preprocessing.

### 3.8. Implementation and Statistics

All models are trained on a single NVIDIA A100 GPU (80 GB). The model is trained in four independent runs with different random seeds, both with the localization loss (λ=0.3) and without it (λ=0). Every main result is reported as the mean ± standard deviation (SD) across runs, with a 95% bootstrap confidence interval (CI) of the run mean over the test images (1000 resamples); for the comparisons with and without the localization loss, the CIs are given in Table A1 and Table A2 and those of the training components in Table A3 (Appendix A). Paired comparisons use the Wilcoxon signed-rank test over test images for measures computed per image, such as AUC-Judd, AIR, deletion and insertion (significant when p<0.05), and a paired bootstrap 95% CI of the AUC and AP difference for detection (significant when the CI excludes zero). A star in the tables marks a difference that is significant in every pair of runs with the same seed. The segmentation baseline is trained in three runs, each compared with the run of our model that uses the same seed. The λ sweep and the component and reduced-mask experiments use one run per setting, compared with our run of the same seed, and their differences are read against the variation across the main runs.

## 4. Results

We report the two training stages, grading and lesion detection, with an analysis of the low microaneurysm result on DDR; then attention as an explanation and its faithfulness; then the ablation and sensitivity analyses; and finally the comparison with a segmentation baseline and training with half of the masks, attention on images with confounding lesions, and the robustness to image degradation.

### 4.1. Stage 1: Grade-Level Pretraining

**Figure 5:**
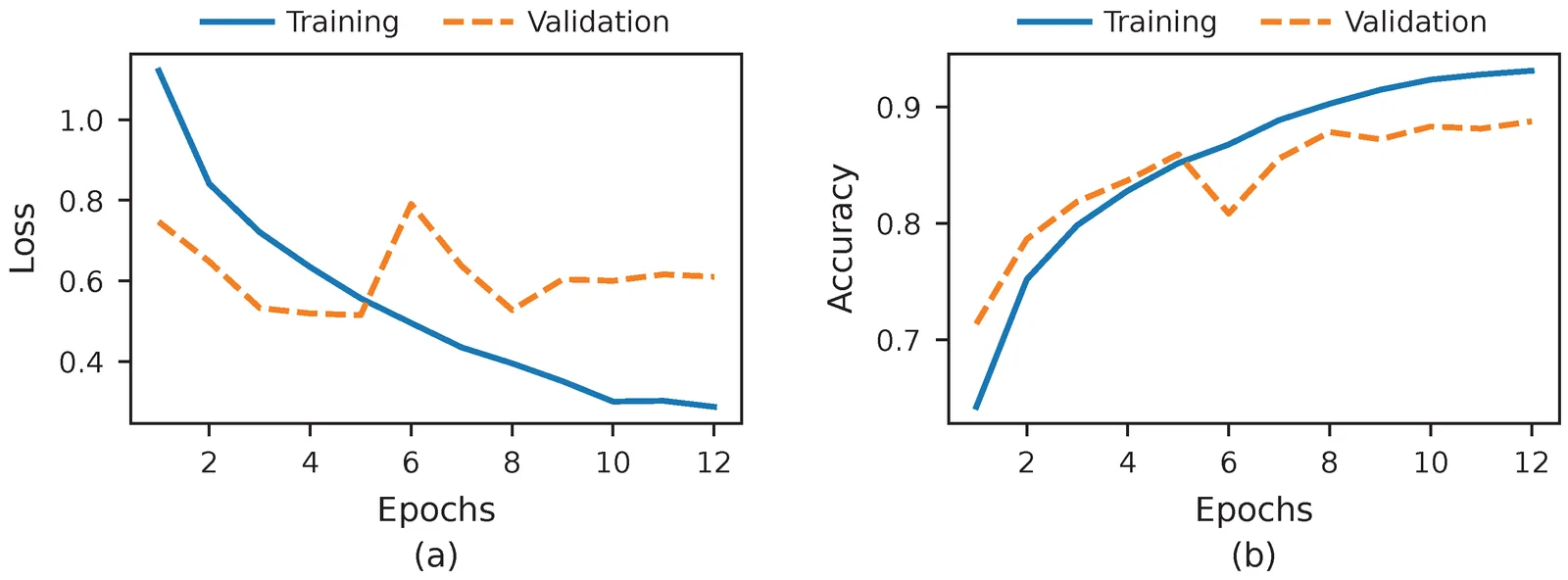
Stage 1 training: (**a**) loss and (**b**) accuracy on the training and validation sets.

**Figure 6:**
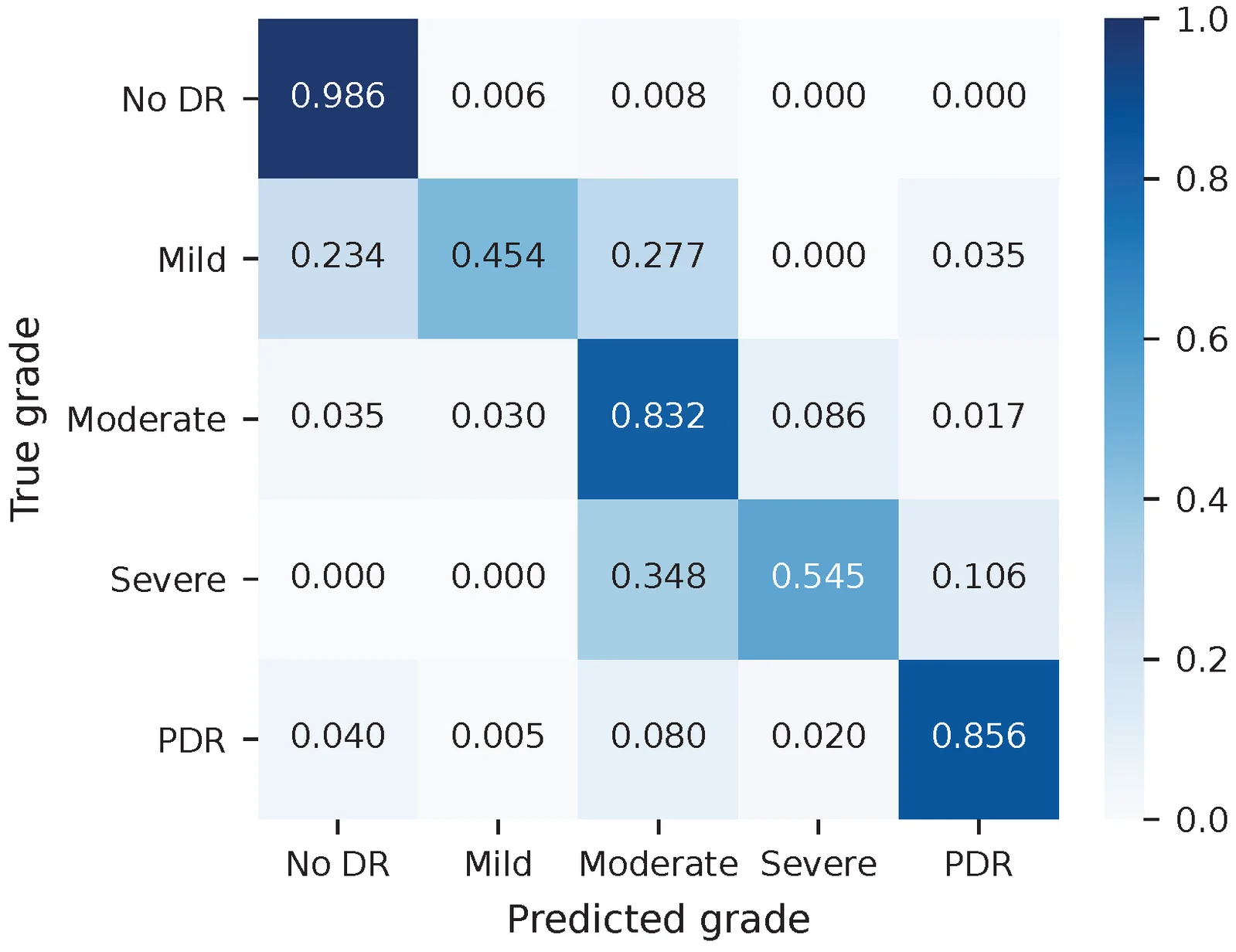
Stage 1 confusion matrix on the validation set; each cell is the share of the images of the true grade.

**Figure 7:**
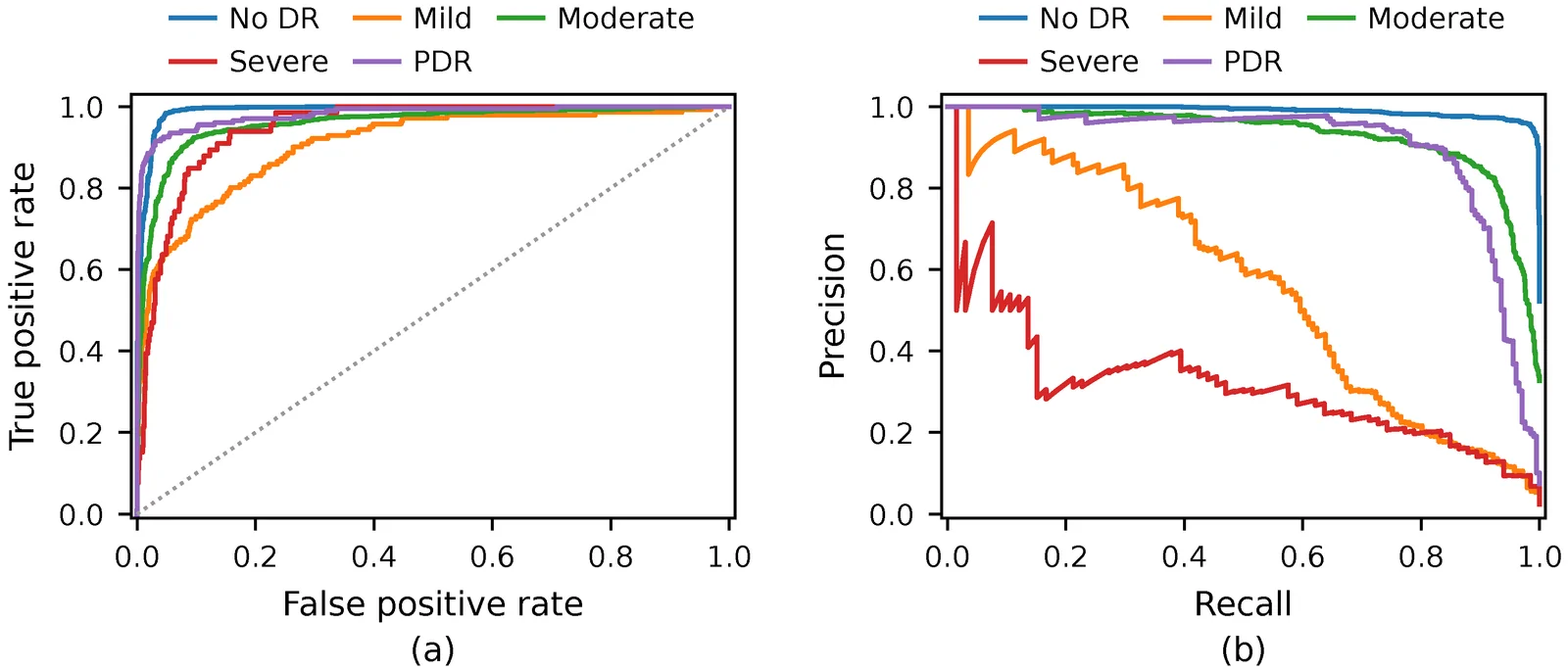
Stage 1 per-grade curves on the validation set, one curve per ICDR grade (0–4): (**a**) receiver operating characteristic (ROC); (**b**) precision–recall.

Stage 1 trains the shared encoder to grade retinopathy severity, giving it a representation of retinal appearance that the lesion queries can build on. Table 4 gives the validation metrics at the best epoch; Figure 5 shows the training curves, Figure 6 the confusion matrix, and Figure 7 the per-grade ROC and precision–recall (PR) curves.

### 4.2. Stage 2: Lesion Detection (All Datasets)

**Figure 8:**
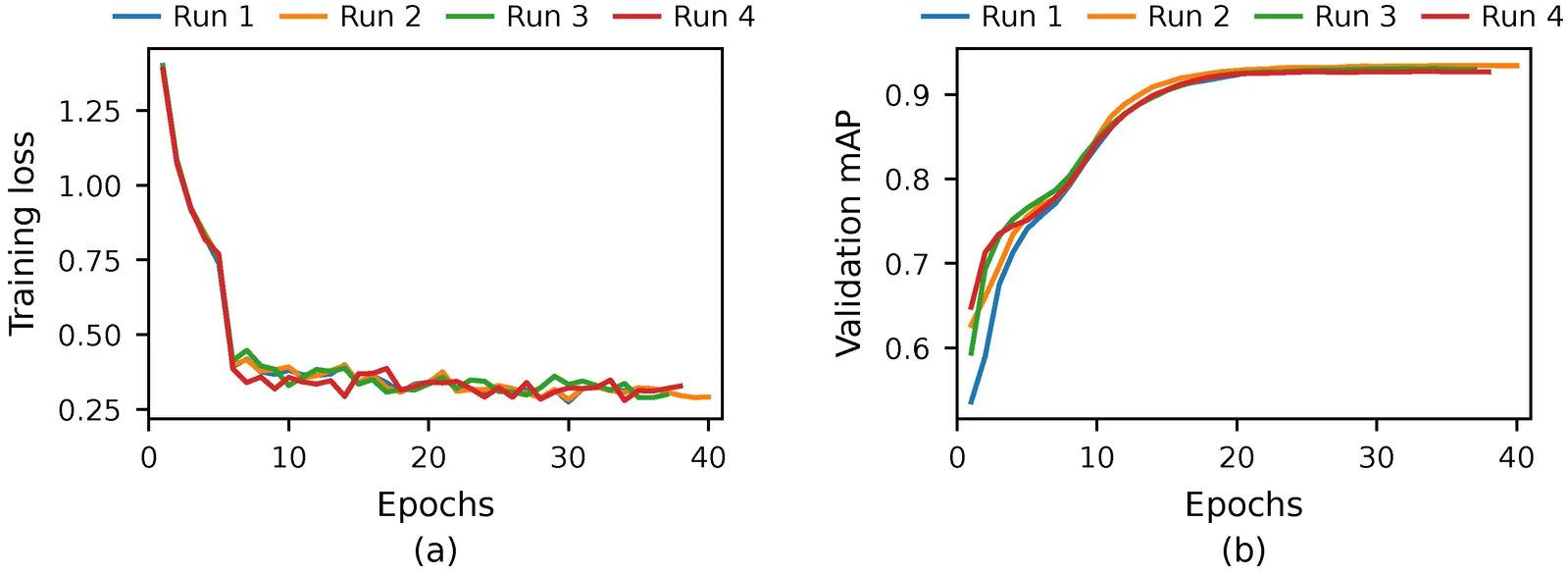
Stage 2 training across runs: (**a**) training loss; (**b**) validation mean average precision (mAP).

**Figure 9:**
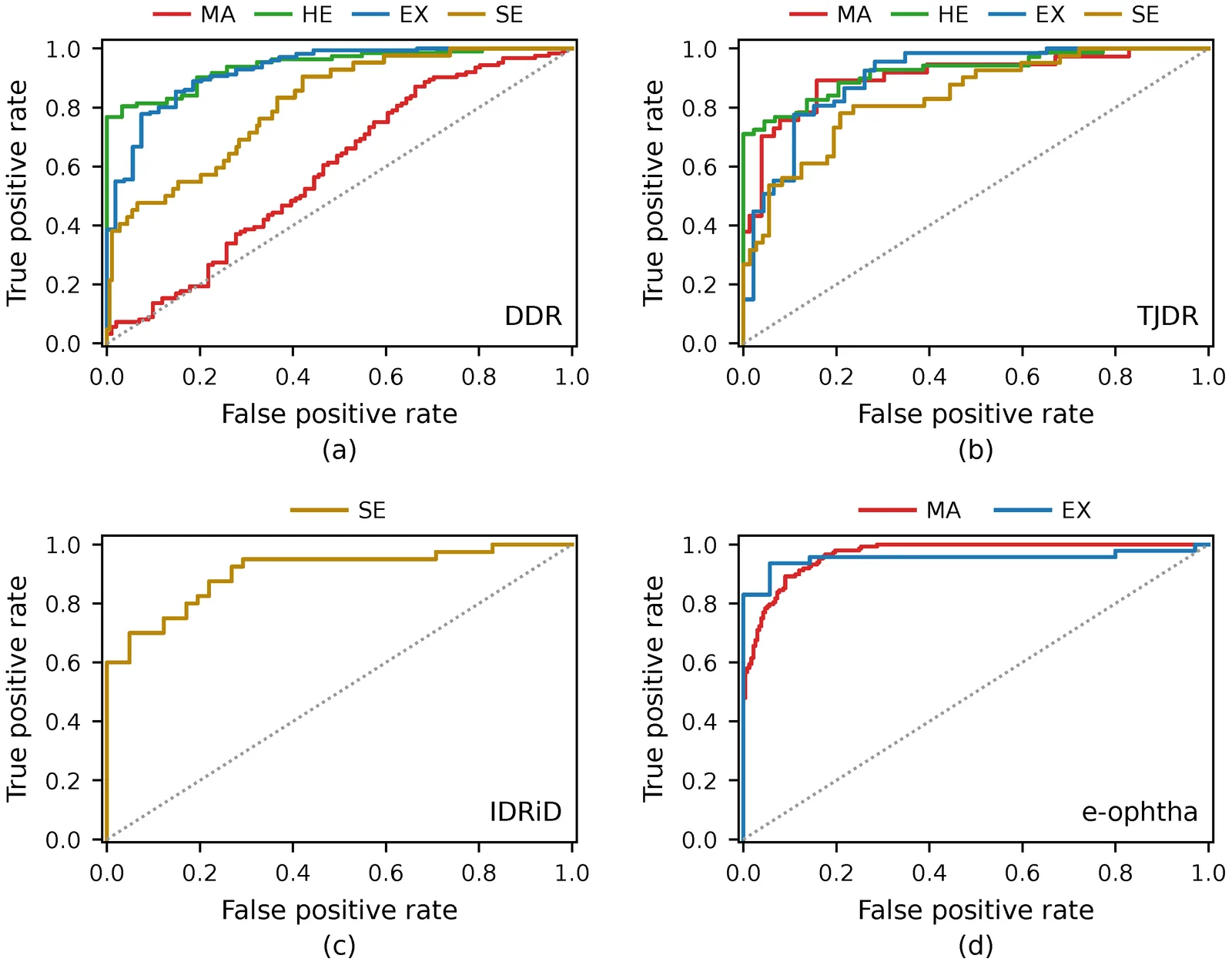
Stage 2 per-lesion receiver operating characteristic (ROC) curves on the test sets for one run: (**a**) DDR; (**b**) TJDR; (**c**) IDRiD; (**d**) e-ophtha. MAs, microaneurysms; HEs, hemorrhages; EXs, hard exudates; SEs, soft exudates. On IDRiD, ROC is not shown for MAs and EXs, which every image contains, or for HEs, which all but one image contains.

**Figure 10:**
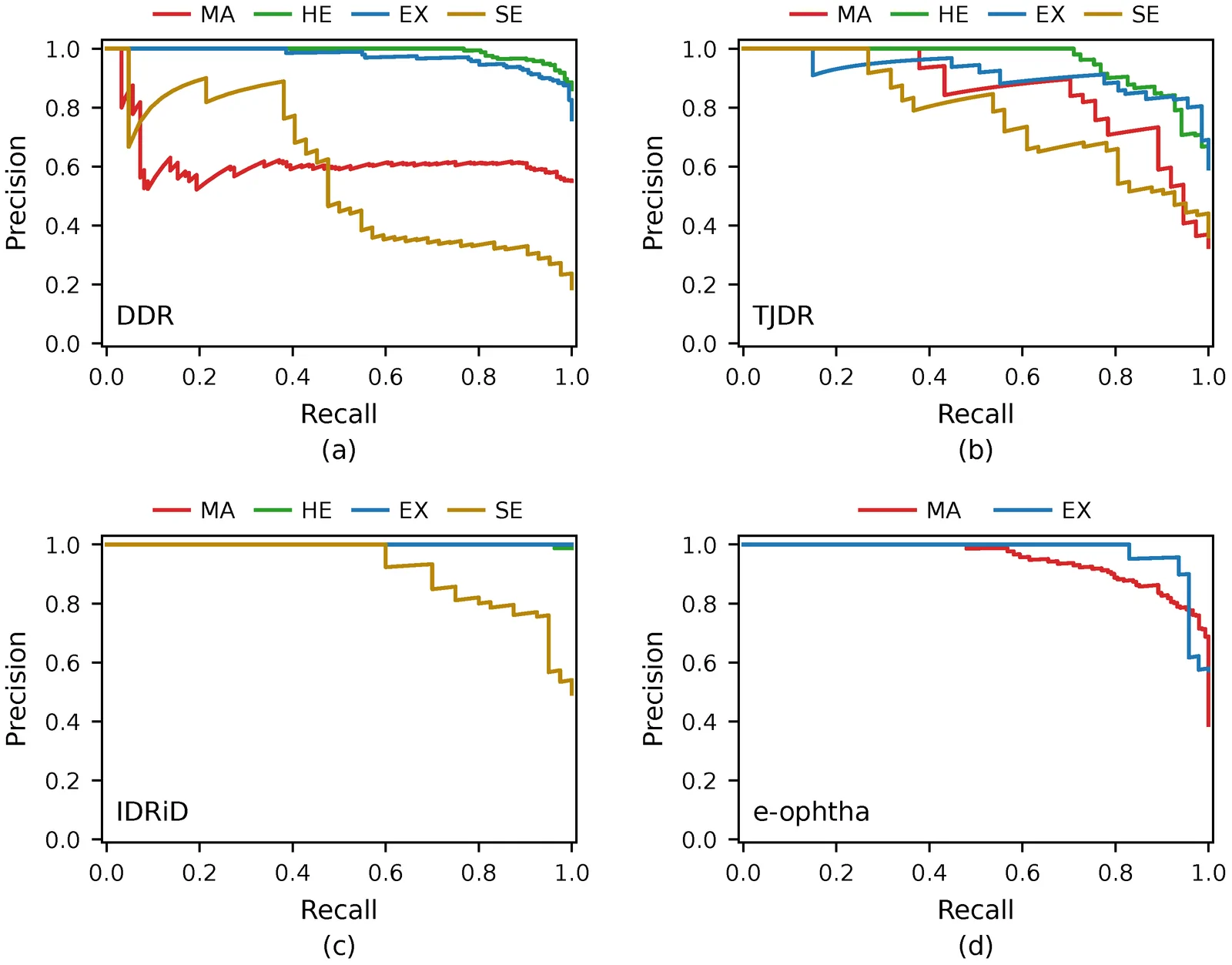
Stage 2 per-lesion precision–recall curves on the test sets for the same run as Figure 9: (**a**) DDR; (**b**) TJDR; (**c**) IDRiD; (**d**) e-ophtha. Lesion abbreviations as in Figure 9.

With the encoder pretrained, we perform fine-tuning for lesion detection (Figure 8). Across the four datasets the model detects the larger lesions well (Table 5; Figure 9 and Figure 10). Hemorrhages and hard exudates are the strongest. On DDR their AUCs are 0.94 and 0.93, with average precision near 0.99 and 0.97, and both are near or above 0.90 on TJDR. Out of domain, e-ophtha, which the model never saw during fine-tuning, reaches AUCs of 0.96 for microaneurysms and 0.96 for exudates against healthy negatives. Because IDRiD and e-ophtha come from different cameras and populations than the DDR/TJDR masks used in training, this suggests that the queries have learned lesion appearance, not one dataset’s particular appearance. On e-ophtha, an MA AUC of 0.96 [87] and exudate AUCs between 0.87 and 1.00 [88] have been reported before, by methods trained on those data and targeting one lesion each.

All values in Table 5 are the means ± SDs across the training runs, with the 95% CIs of the AUC and of the AP.

Microaneurysms on DDR reach an AUC of only 0.58, far below every other entry; the cause lies in the composition of the DDR MA-negative class, which Section 4.3 examines. Soft exudates are the other weak point: SE is the rarest lesion in the training set, and its F1 and average precision are lower throughout. This is why we read average precision alongside AUC, since the former is more informative under heavy class imbalance [89]. With only 42 positive images in the DDR test set, its AUC there carries a wide 95% CI (0.740–0.878). We summarize in-domain performance with macro mAP (DDR, 0.79; TJDR, 0.88). We do not report a single mAP for IDRiD: microaneurysms and exudates are present in all 81 of its images and hemorrhages in all but one, so their average precision is trivially high, the AUC is undefined or not informative, and averaging these into a dataset-level figure would inflate it. We therefore report IDRiD per lesion only, as a check of generalization. The e-ophtha set labels only two of the four lesions, so a four-lesion mAP is not defined there either.

### 4.3. Microaneurysms: Healthy Versus Confounding Negatives

The low microaneurysm AUC on DDR could come from the lesion itself or from the images that it is compared against. DDR alone cannot separate the two: in total, 100 of the 101 DDR test images without microaneurysms contain hemorrhages, and none is free of lesions. We therefore score microaneurysm detection against each group of MA-negative images separately (Section 3.5 and Table 6). Detection stays high against images with no lesions or with exudates only and drops against images with hemorrhages: slightly on TJDR (0.866) and sharply on DDR (0.580). On DDR, images with hemorrhages receive almost the same microaneurysm score as images with microaneurysms (mean probability of 0.807 against 0.857).

What lowers the result is not hemorrhage in general but small hemorrhages that resemble microaneurysms (Table 7). The DDR test images without microaneurysms contain 2322 hemorrhage spots no larger than most microaneurysms, against 40 in TJDR, and in DDR, these spots have the same diameter and contrast in the photograph as the microaneurysms themselves. The microaneurysm attention is drawn to them: on the DDR images without microaneurysms, 4.1% of the microaneurysm attention falls on hemorrhages that cover 1.4% of the retina.

Two further checks support this. The size of the lesion is not the cause: no MA-positive image in either test set loses all of its microaneurysm mass when the mask is max-pooled to the token grid, even at 16×16, because a positive image carries a median of 8.5 separate components on DDR and 3 on TJDR. Nor can the reference masks alone separate the two classes on DDR. An MA-negative DDR image carries a median of 26 hemorrhage components, more than half (median 56%) of them within the microaneurysm size range, against none in half of the MA-negative TJDR images, and ranking each test image by the number of red components within the microaneurysm size range, read from each set’s own reference masks rather than from any model output, gives AUCs of 0.491 on DDR and 0.952 on TJDR.

### 4.4. Attention as Explanation

**Figure 11:**
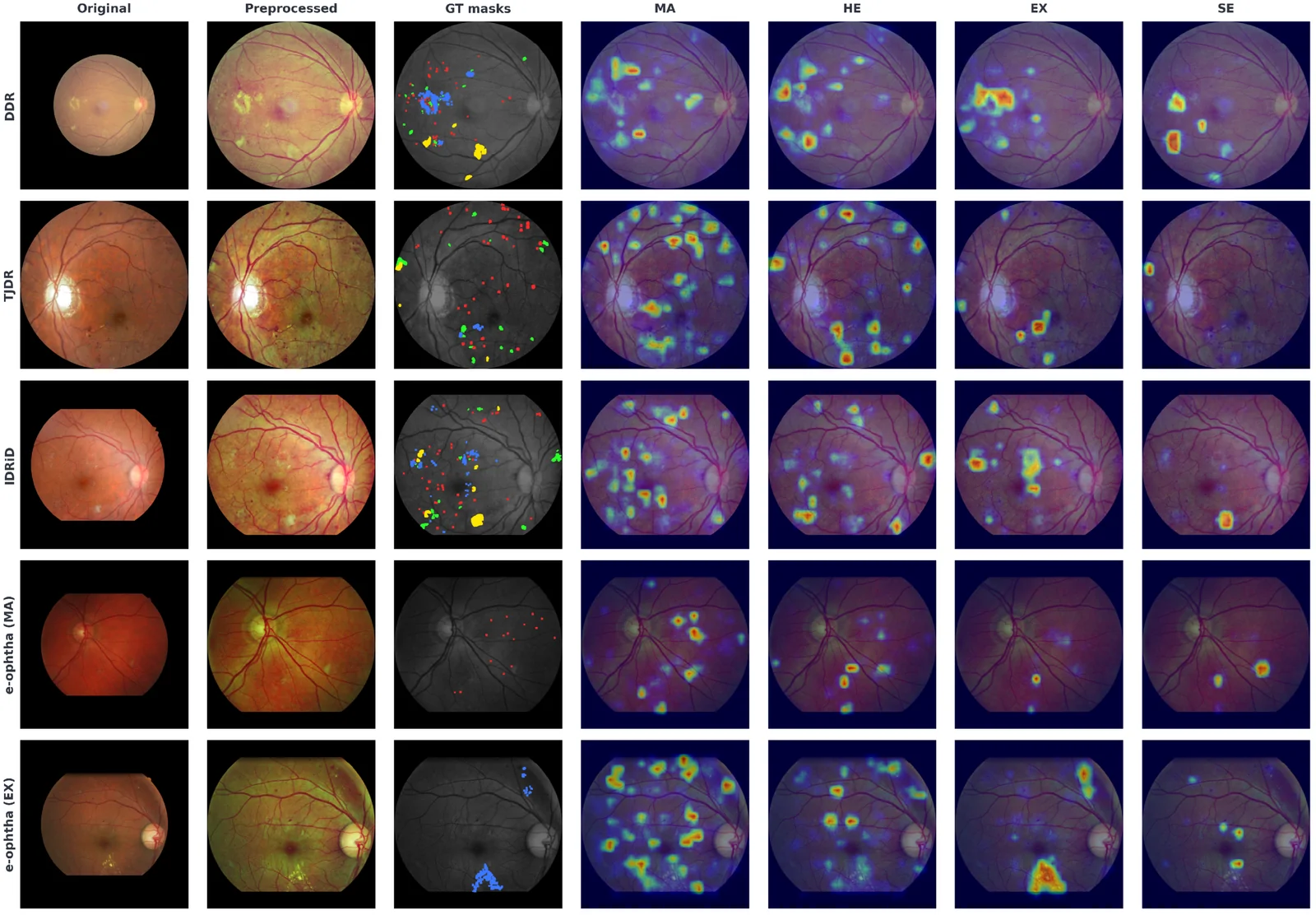
Per-lesion attention on test images. Rows: one image each from DDR, TJDR, IDRiD, and the e-ophtha MA and EX subsets. Columns: original image; preprocessed image; ground-truth (GT, reference) masks (MAs in red, HEs in green, EXs in blue, and SEs in yellow); and the attention map of each lesion query overlaid on the image, with warmer colors indicating higher attention.

Besides the detection scores, the model gives one attention map per lesion. Since each lesion has its own query, each attention map is the explanation for that lesion. We read these maps directly, without computing a separate explanation after training. On test images from all four datasets, each query lands on its own lesion: the EX query on hard-exudate clusters, the HE query on hemorrhages, and so on. In the examples shown, this also holds on IDRiD and e-ophtha, which the model never saw during fine-tuning. So the four queries do not share one map; each answers for its own lesion (Figure 11).

**Figure 12:**
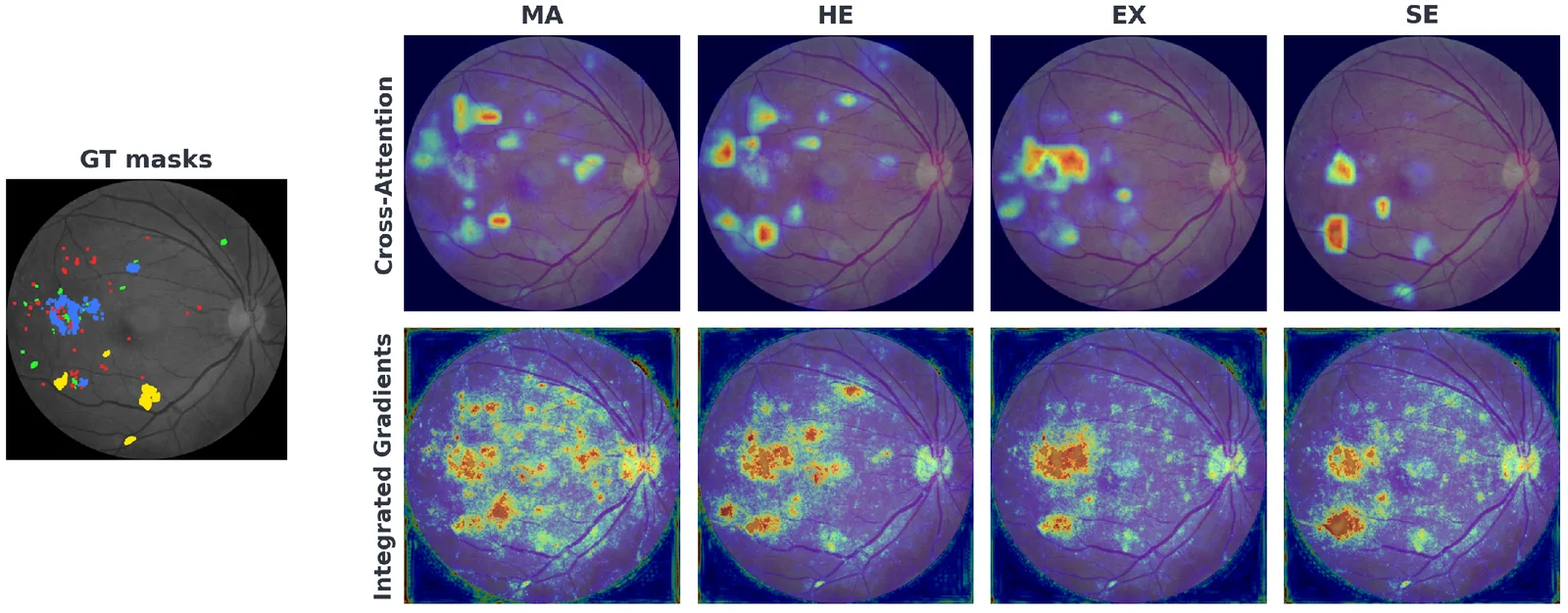
Cross-attention (top row) versus Integrated Gradients (bottom row) for each lesion query on a DDR test image; left, ground-truth (GT, reference) masks: MAs in red, HEs in green, EXs in blue, and SEs in yellow. Warmer colors indicate higher attention or attribution.

**Figure 13:**
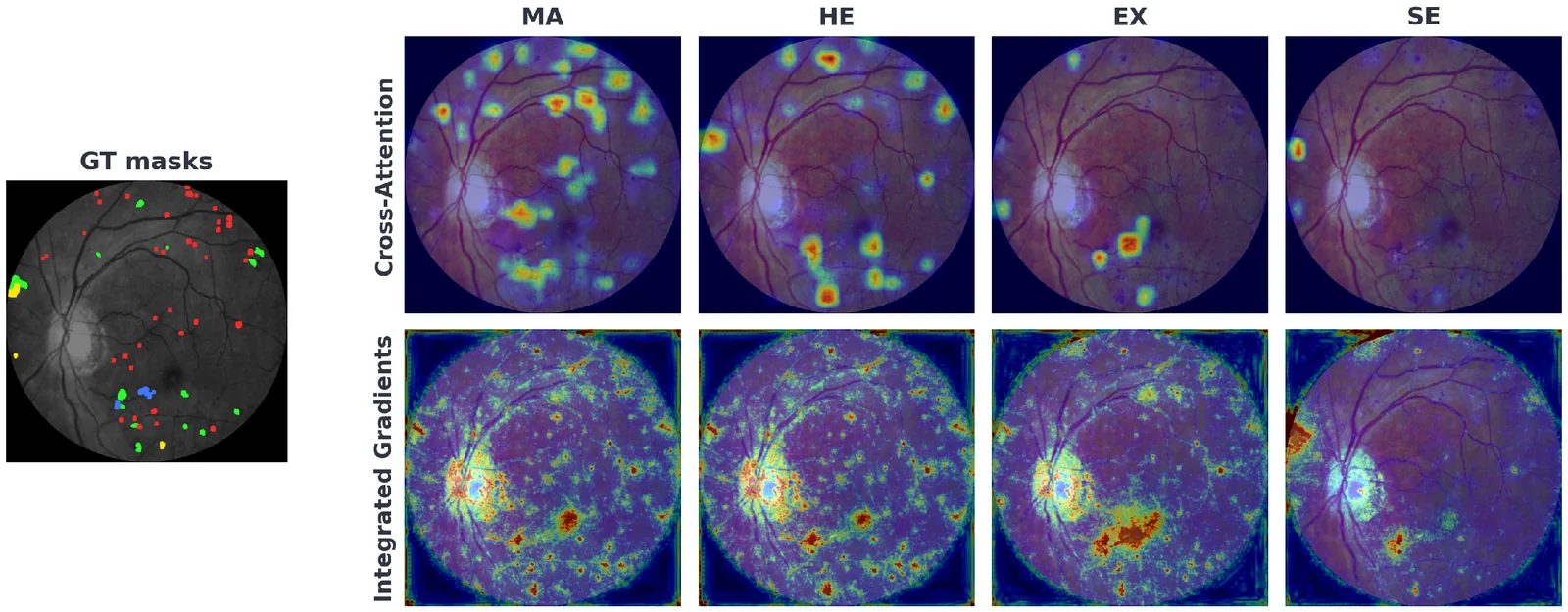
Cross-attention (top row) versus Integrated Gradients (bottom row) for each lesion query on a TJDR test image; left, ground-truth (GT, reference) masks: MAs in red, HEs in green, EXs in blue, and SEs in yellow. Warmer colors indicate higher attention or attribution.

We compare attention with Integrated Gradients (Section 3.6). A DDR and a TJDR test image show the difference (Figure 12 and Figure 13): cross-attention forms tight spots inside the retina, while the Integrated Gradients map is less focused and leaks onto the black border outside the retina.

AUC-Judd measures whether attention ranks lesion pixels above the rest of the retina, on the DDR and TJDR test sets (Table 8). Every lesion on both datasets scores between 0.948 and 0.996, against a chance value of 0.5, with a standard deviation of at most 0.002. Soft exudates rank the highest on both datasets, and microaneurysms reach 0.952 on DDR and 0.949 on TJDR despite covering a median of 0.027% and 0.017% of the image. The two datasets agree to within 0.007 for three of the four lesions. For microaneurysms, attention is as accurate on DDR as on TJDR, so their lower detection on DDR (Section 4.3) does not come from where the model looks.

### 4.5. Faithfulness: Deletion and Insertion

A map that looks right must also be shown to drive the prediction, and the deletion and insertion test supports that it does (Table 9). Deletion AUC is low across lesions on both DDR and TJDR, about 0.50 for microaneurysms (0.495 and 0.483), and insertion AUC is high, above 0.80 for MA and 0.89 for hemorrhages on DDR. Soft exudates are the weakest on insertion, consistent with their rarity and the lower detection scores already seen, but even there, deletion stays the lowest of the four lesions. For hard exudates, the blurred image that insertion starts from already keeps much of the bright exudate evidence, so restoring the attended pixels raises the probability less.

### 4.6. Ablation and Sensitivity Analyses

We report, in turn, the effect of removing the localization loss, of its weight, and of removing each of the other training components (Section 3.7.1).

#### 4.6.1. Localization Loss

*Detection.* Adding the localization loss does not reduce detection; mean mAP is slightly higher with the loss in every run. Test-set macro mAP moves from 0.779 to 0.792 on DDR and from 0.855 to 0.878 on TJDR, and the per-lesion AUC and AP shift by comparably small amounts (Table 10).

**Figure 14:**
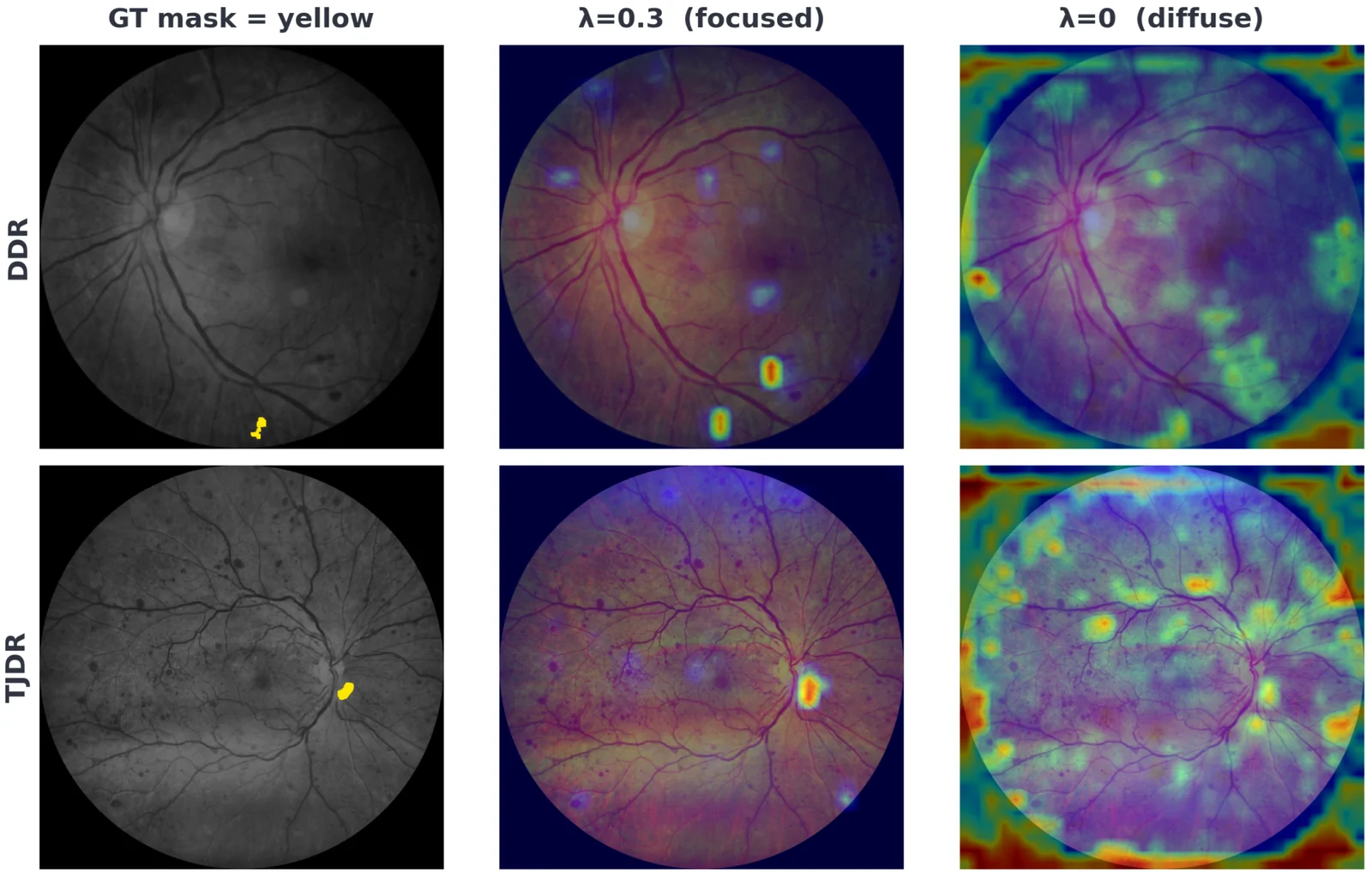
SE-query attention with and without the localization loss (λ = 0.3 vs. λ = 0) on one DDR and one TJDR test image; left, ground-truth (GT, reference) soft-exudate mask (yellow).

*Attention on the retina.* Where attention falls, by contrast, changes markedly, most of all for the soft-exudate query. With λ = 0.3, its attention sits on the lesion and stays inside the retina; with λ = 0, it spreads out and reaches the black area around the retina (Figure 14). AIR measures how much of the attention is inside the retina. Without supervision it is 0.69 to 0.84; with supervision it rises to about 0.99, for all four lesions on both datasets (Table 10). The biggest rise is soft exudates on DDR, up by 0.31. These gains, from 0.16 to 0.31, are seven to twenty-three times larger than either change in mAP, so the loss acts mainly on the explanation and improves detection slightly.

*Localization and faithfulness.* AIR shows that attention stays on the retina but not that it falls on the lesion. We therefore also compare AUC-Judd and the deletion/insertion test with and without the loss (Table 10 and Table 11). With the loss, AUC-Judd rises for every lesion on both datasets (Wilcoxon signed-rank test, p<0.05), and its spread across runs falls from up to 0.141 to at most 0.002 without the loss, where attention lands is not stable. With the loss, attention also marks the regions that the model’s score depends on: removing the most-attended pixels lowers the probability more, for every lesion on both datasets (lower deletion and higher drop), significantly for five of the eight. Restoring them onto a blurred image raises it more for microaneurysms, hard exudates and soft exudates (higher gain). The raw insertion, however, is higher without the loss for hard exudates and for hemorrhages on DDR, as is the gain for hemorrhages on DDR. This higher insertion without the loss does not come from the lesion. In the first 10% of restored pixels (Table 12), the map trained with the loss restores 82–94% of the lesion area, against 56–81% without it, and up to 24% of the restored pixels fall outside the retina without the loss, against about 1% with it. This holds on both datasets and also when 20% of the pixels are restored (Wilcoxon, p<0.01). For hard exudates, the model without the loss also starts from a higher probability on the blurred image. Deletion, which removes only the attended pixels, favors the loss for both lesions.

#### 4.6.2. Loss Weight λ

Any value of λ above zero improves attention clearly (Table 13). From 0.1 to 1.0, AUC-Judd is stable (DDR, 0.964–0.969; TJDR, 0.980–0.981), and the pixel AP of every value from 0.1 to 1.0 falls within the range of λ=0.3 across its training runs (DDR, 0.138–0.148; TJDR, 0.143–0.160), so the conclusions do not depend on the exact value. λ=0.3 lies within this stable range and gave the highest validation mAP.

#### 4.6.3. Training Components

Of the components removed (Table 14), Stage 1 pretraining has the largest effect on detection, and the asymmetric loss helps mainly for hemorrhages on TJDR; elsewhere the differences are small and mostly not significant. Without MixUp, the soft-exudate AUC is higher (DDR +0.031, significant; TJDR +0.017), and the other lesions change by 0.01 or less in either direction. The weight average has no consistent effect.

Apart from Stage 1 pretraining, which gives the encoder its general retinal features, the components hardly change the attention maps: the mean AUC-Judd of the four lesions stays between 0.961 and 0.969 on DDR and between 0.979 and 0.981 on TJDR for every other variant (0.967 and 0.980 with all components), against 0.951 and 0.977 without Stage 1. Their quality comes mainly from the localization loss.

### 4.7. Comparison with a Segmentation Baseline and Training with Half of the Masks

Against U-Net (Section 3.7.2), our model detects lesions better on average (Table 15): its mean AUC is 0.876 against 0.853. Our model is significantly better for hard exudates on DDR, microaneurysms and hemorrhages on TJDR, and microaneurysms on e-ophtha; U-Net is higher for microaneurysms and soft exudates on DDR, soft exudates on IDRiD and hard exudates on e-ophtha, but none of these differences is significant, although for soft exudates on IDRiD and hard exudates on e-ophtha, U-Net is higher in every pair of runs. For localization (Table 16), U-Net has a much higher pixel AP for every lesion: it is trained to classify each pixel against the mask and outputs a 512×512 map, whereas our attention is a 32×32 map trained to match the distribution of the mask, so it marks the lesion region without tracing its outline. On AUC-Judd, which measures whether the lesion region is ranked above the rest of the retina, U-Net is higher for microaneurysms and hard exudates and our model for hemorrhages and soft exudates (significant on DDR for microaneurysms and hemorrhages, respectively), and our maps vary less between runs (SD at most 0.002, against up to 0.017).

Unlike U-Net, which can learn only from images with masks, our model also learns detection from the image-level labels of the images without them. With masks on only half of the training images (Table 17), the attention maps stay essentially the same (AUC-Judd values of 0.964 against 0.967 on DDR and of 0.980 for both on TJDR), and since detection is trained from the labels of all 832 images, the mean detection AUC over the eleven lesion–dataset pairs of Table 15 is unchanged (0.874, as for the run with all masks and the same seed), still above U-Net trained on all the masks (0.853; Table 15). On the external sets, the detection AUCs are 0.905 for soft exudates on IDRiD and 0.970 for microaneurysms and 0.957 for hard exudates on e-ophtha. Per lesion, the AUC is slightly higher with half of the masks for some lesions and slightly lower for others, so the mean is unchanged.

### 4.8. Attention on Images with Confounding Lesions

**Figure 15:**
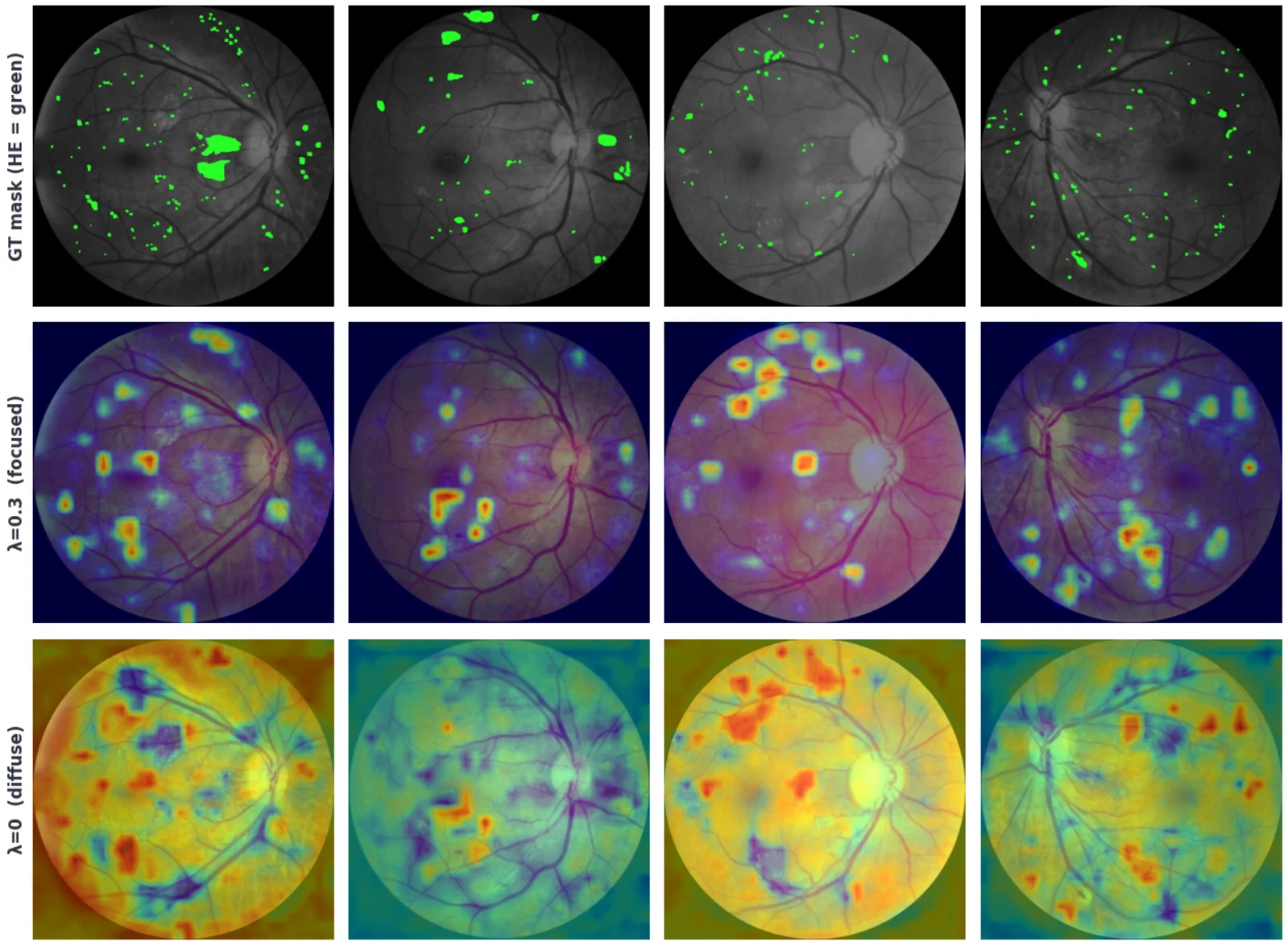
Microaneurysm-query attention on four DDR test images without microaneurysms that contain hemorrhages. Top row: ground-truth (GT, reference) hemorrhage mask (green); middle row: with the localization loss (λ=0.3); bottom row: without it (λ=0).

On test images that lack the target lesion but contain other lesions (Section 3.7.3), the query trained with the localization loss, having learned what its lesion looks like, is drawn partly toward similar-looking lesions (Figure 15), but only with 1.6–5.9% of its attention across lesions and datasets, more than the 0.2–2.3% without the loss (Table 18).

This attraction does not lower detection compared with the model trained without the loss: on the same images, the model trained with the loss separates images with the lesion from images with other lesions about as well as or better than the model trained without it; for hemorrhages on TJDR, for example, the AUC is 0.919 against 0.847 (Table 18).

### 4.9. Robustness to Image Degradation

On the altered test images (Table 19), changes in brightness and contrast change the mean AUC by at most 0.012, and blur and strong JPEG compression lower it by 0.03 to 0.07. Sensor noise causes the largest drop, for every lesion (tied with JPEG compression for DDR soft exudates) and most of all on TJDR, where the mean AUC falls to 0.539, close to chance. The model never saw added noise during training: noise and blur were not added as augmentation, because they bury microaneurysms, the smallest lesion. Robustness to different cameras and sites is supported by the external datasets, IDRiD and e-ophtha, which other teams collected with different cameras and which were never used in training (Table 5).

## 5. Discussion

Our model detects lesions well on e-ophtha (AUC of 0.96 for microaneurysms and exudates) and soft exudates on IDRiD (0.90), sets that it never trained on and which other teams captured and labeled with different cameras. If the model had only memorized one dataset’s style (its camera, lighting, and labeling), it would not work this well on the others. These external sets are, however, small, and e-ophtha labels only two of the four lesions; we therefore read them as supporting evidence of generalization rather than as proof.

The main exception is microaneurysms on DDR: their AUC of 0.584 sits far below the other lesions, even though their attention lands on microaneurysms as precisely as on TJDR (AUC-Judd of 0.952 against 0.949). Section 4.3 traces this to DDR’s MA-negative images rather than to the size of the lesion: nearly all of them contain hemorrhages, many as small as microaneurysms, and even the reference masks cannot separate the two classes there (AUC 0.491). This confusion is not specific to the model: a microaneurysm and a similar-sized dot hemorrhage are both small round red spots, and ophthalmologists themselves cannot reliably tell the two apart on a color photograph without fluorescein angiography [90]. Doing so needs a different modality. Fluorescein angiography fills a microaneurysm with dye but requires an intravenous injection; structural optical coherence tomography (OCT) and OCT angiography image the wall of a microaneurysm and the flow inside it with no dye at all [91]. Pan et al. report an AUC of 0.94 for microaneurysms on fluorescein angiography, where the dye makes them easy to see [92]. None of these are available for the fundus photographs that our datasets are built from. Clinically, the distinction matters less: He et al. surveyed ophthalmologists and concluded that distinguishing a microaneurysm from a similar-sized dot hemorrhage is not necessary for DR grading [90]. A pixel-level model faces the same material: RTNet reports 0.12 AUPR for microaneurysms on DDR against 0.49 on IDRiD [54], a gap that sparsity alone does not explain, since microaneurysms are equally sparse in both. Still, the model’s 0.584 is above the 0.491 that the reference masks reach on the same images.

Soft exudates are the other difficult lesion, and here the problem is prevalence, not size: SE is the rarest lesion in the training set, and the resulting class imbalance shows up as lower F1 and average precision even where AUC is respectable. For both lesions, the mask supervision improves where the model looks, clearly so for SEs. But it does not fix the root problems, the composition of the DDR MA-negative class and the rarity of SEs, which limit how well they can be told apart.

Scoring attention only against the same masks that it was trained on would mostly reward the loss for what it already does, so we also use two measures that do not need the lesion masks. AIR asks something that the training does not directly aim for: whether the attention is inside the retina at all. And it needs no masks, so it also works for microaneurysms: they are only about five pixels across at 1024×1024 (diameter in Table 7), while each attention cell covers 32×32 pixels, so a shift of a few pixels in the mask changes any overlap with it a lot. The deletion and insertion tests show the same thing another way: the regions that the attention marks are the ones that the prediction depends on, and less so without the loss (Table 11).

Without the localization loss, detection is slightly lower while attention scatters, in part onto the black border (Section 4.6.1). We read this as a split of roles. It is consistent with the encoder’s self-attention, which lets every token carry information from the whole image, so a query can read the evidence for a lesion from tokens far from it; with the loss, the query reads from the lesion itself. The classification loss thus handles the prediction and the localization loss the explanation, and this split is this paper’s main finding. It also answers the open problem that Papadopoulos et al. [28] left: whether pixel masks can sharpen an attention map that already localizes lesions on its own.

**Comparison with segmentation.** Our framework also needs pixel masks, and image-level scores can be read from a segmentation map, so the question is what the per-lesion design adds. The comparison with a U-Net trained under the same conditions (Section 4.7) points to two practical differences. First, the image-level decision is trained directly as a per-lesion output rather than derived from a pixel map by a separate rule, and it detects lesions better. Second, supervision can be mixed: a segmentation model learns only from images that have masks, whereas ours also learns detection from the labels of the images without them; with masks on only half of the training images, our model keeps its detection and stays above U-Net trained on all the masks. When the exact extent or number of lesions is needed, a segmentation model is the better choice, since it outlines lesions more precisely (Table 16); its finer map (512×512 against 32×32) is also consistent with its higher AUC-Judd for microaneurysms, the smallest lesion. We therefore see the two approaches as complementary.

**Intended use of the attention maps.** The maps are meant to support an ophthalmologist. For each lesion type that the model reports, the ophthalmologist can go straight to the highlighted region, confirm or reject the finding, and spend less time searching the whole image. Because each lesion type has its own map, the ophthalmologist can also see which region a microaneurysm score rests on, including regions that the reference masks label as hemorrhage (Figure 15). Each map cell covers a 32×32-pixel block of the 1024×1024 input, so the map shows the neighborhood that contains a lesion, not its outline: it reflects how attention weights are distributed, not anatomical boundaries, and should not be read as a segmentation mask or used to count lesions or measure their extent.

**Mask reliability.** We do not treat the absence of an annotation-specific check for DDR, TJDR, and e-ophtha as evidence that their masks are accurate. A re-check exists only for IDRiD, where an independent review found classification errors and missing data: it raised the hard-exudate area by about half and removed artifacts that had been labeled as hemorrhage. Even after expert correction, the raters still agreed at only F1 = 0.90 [93]. All four datasets use the same color fundus imaging and the same manual annotation process, done mostly in a single pass, so we consider that they carry comparable classification flaws. We therefore treat the masks of all four sets as a useful but imperfect reference, and we read our per-lesion and mask-agreement results in that light; when the model disagrees with a mask, the mask is not necessarily correct. In line with this, TJDR needed a third senior ophthalmologist to reconcile regions where two raters disagreed [14], and DDR provides pixel masks for only a curated subset of its images [11].

Four factors explain why the masks are imprecise. *(i) Imaging modality:* Color fundus photographs show only a small share of the microaneurysms that fluorescein angiography reveals; they detected only 4.12% of them in a recent study [94] and 6.6% 25 years earlier [95], so many real lesions cannot appear in any mask. *(ii) Under-annotation:* Small and faint lesions are missed in a consistent way, a bias strongest for microaneurysms, which cover only about 0.1% of the pixels in a fundus image [96]. *(iii) Inter-class confusion:* The two red lesions (MAs and HEs) are hard to separate on color photographs, and the annotators of TJDR note that small hemorrhages resemble microaneurysms in color and morphology [14], to the point that the e-ophtha microaneurysm subset is described as containing “microaneurysms or small hemorrhages” [13], and hard exudates are prone to being misclassified as cotton-wool spots, that is, as soft exudates [97], so some mask labels are attached to the wrong class. *(iv) Coverage gaps:* Only four non-proliferative lesions are annotated, leaving proliferative findings (neovascularization, intraretinal microvascular abnormalities (IRMA), and vitreous hemorrhage) unlabeled [93], so a region marked negative may contain real, unlabeled disease.

One more factor concerns the input that the model actually sees. The public masks are drawn on the original photographs, while our model works on CLAHE-enhanced input, and CLAHE enhances contrast, which the authors report is helpful for microaneurysm detection [73]. The annotation viewing conditions are not reported, so we cannot say whether the graders used any enhancement, but the model likely has a contrast advantage over the original grading view. So for the detections that the model makes outside the masks, we separate three cases: (a) real lesions that CLAHE reveals but that were too faint for the annotator or were otherwise missed, where the model is right and the mask is incomplete [98]; (b) noise or look-alike spots that CLAHE made stand out with no real lesion underneath; and (c) real but non-target structures in the image. We do not claim that all these detections are true lesions, and we cannot separate the cases without re-annotation. Part of the apparent disagreement may therefore come from the limits of the annotations, not only from the model.

Finally, our design is not very sensitive to this label noise, for two reasons. First, the localization loss is soft: it keeps each query’s attention on the lesion area in general, instead of forcing it to match the exact mask boundary. Second, two of our measures use no masks at all, AIR and the deletion/insertion test, so our main faithfulness results do not need perfect masks. Measuring exactly how much of the disagreement comes from labeling would need a more accurate reference, such as fluorescein angiography, structural OCT or OCT angiography, or a multi-grader consensus, which we leave to future work.

**Limitations.** The attention maps show where the model attends, evaluated against the available annotations and by perturbation tests; they are lesion-aligned evidence, not definitive causal explanations of the model’s decisions. Robustness to sensor noise is limited (Table 19): noise and blur were not added as training augmentation, to protect microaneurysms, so the model was not trained on images with added noise. The maps are 32×32 because attention reads the last stage of the backbone; a finer map, from a larger input or from an earlier stage with four times as many tokens, multiplies the attention cost about sixteenfold. Finer maps, which would sharpen the localization rather than the detection, are left for future work.

Three directions could extend this work. The first is data: The training data could cover lesions beyond the four we studied, and images that carry image-level lesion labels alone. Image-level lesion labels are far cheaper to collect than pixel masks, and since the model keeps its detection with masks on half of the training images (Section 4.7), a small mask-supervised set could be paired with a much larger labeled one. How small the mask-supervised set can be and whether this holds on other datasets need further investigation. The second is preprocessing and augmentation: We use CLAHE on the green channel, but adaptive contrast enhancement on the L channel of LAB color space keeps the lesion colors and adds less noise [99]. Because it preserves the yellow and pale look of hard and soft exudates, it may help with those two lesions. We leave a green-versus-LAB comparison to future work, together with noise augmentation at levels that keep microaneurysms visible, which may improve robustness to sensor noise. The third is clinical: a study with ophthalmologists, measuring accuracy and reading time with and without the maps, is needed to establish their diagnostic utility.

## 6. Conclusions

In this work we built a diabetic retinopathy lesion detector with a separate attention query for each lesion, so that one model returns a score and an attention map for every lesion in a single pass and each map is trained on that lesion’s own mask. Without reducing detection, the localization loss moves attention onto the lesions, so the explanation is learned during training rather than derived after it. Attention falls on the lesions, and the deletion and insertion tests support that the prediction depends on it: removing the attended pixels lowers the lesion probability, and adding them back raises it. Where detection is the hardest, on microaneurysms in DDR, the drop comes from the MA-negative images, nearly all of which contain hemorrhages, rather than from lesion size. These findings hold across localization weights from 0.1 to 1.0. Compared with a U-Net trained under the same conditions, the model detects lesions better at the image level and, unlike it, also learns from images without masks, keeping its detection when only half of the training images have masks, while U-Net outlines lesions more precisely; the maps are therefore best used to guide the ophthalmologist to each reported lesion rather than to measure it.

## Figures and Tables

**Table 1:** Representative methods in DR grading, lesion detection, localization, and segmentation.

Method	Task	Mechanism	Explanation	Datasets	Key Limitation
GREEN [38]	DR grading	GCN over five grade nodes; prior frozen at test time	Class-dependency matrix, before and after training	DRD and APTOS2019	No lesion representation
CABNet [37]	DR grading	Per-grade category block and class-agnostic global block	Grad-CAM heatmaps of attention-module outputs	DDR, Messidor, and EyePACS	Localizes coarse regions but not lesion type; authors leave this to future work
SSiT [39]	DR grading	Saliency drops low-salience patches in momentum contrast	Self-attention maps	EyePACS (pretrain); DDR, Messidor-2, and APTOS	Saliency hand-computed; it shapes pretraining only
LQAP [22]	DR grading	Lesion-query cross-attention pooling with query self-attention (8 queries); ordinal evidential head	Query activation maps	APTOS, Messidor-2, and EyePACS	Queries do not map to named lesions (as the authors note); no lesion output; preprint
RTNet [54]	Pixel-level segmentation	Global Transformer block; lesion–lesion and lesion–vessel relation block	Query-specific attention maps	IDRiD, DDR	Dense masks needed; relies on coarse vessel pseudo-masks
HACDR-Net [57]	Pixel-level segmentation	Heterogeneous convolutions; attention aggregation; Noise Adjusted Loss	Grad-CAM over encoder branches; t-SNE of features	DDR and IDRiD	Dense masks needed; kernel size trades MA accuracy for overall performance
Cheng et al. [49]	Multi-label lesion detection	CNN features re-weighted by a GCN over label co-occurrence	—	Self-collected (7459 images)	No localization; label set mixes lesions with non-lesion findings
Jiang et al. [50]	Multi-label lesion detection	Fully convolutional ResNet-50 with post hoc Guided-Grad-CAM	Guided-Grad-CAM thresholded into lesion contours	Private set (3228 images)	Five labels; attention post hoc and unsupervised
Cross-domain WSL [44]	Multi-label lesion detection	Lesion-patch MIL (LpMIL) with semantic-constraint adaptation (LpSCA)	—	FGADR (source), EyePACS (target), and EyePACS-pixel (eval)	Patch-level output only; coarse attribute labels; HE, SE, and EX only
Quellec et al. [41]	Referable-DR detection + lesion detection	ConvNet trained on referable-DR labels; hue-preserving sensitivity map with a sparsity term optimized during training	Single-channel sensitivity heatmap (FROC at lesion level; ROC at image level)	Kaggle DR, DiaretDB1, and e-ophtha (private)	One heatmap per binary output; per-lesion separation from separate training checkpoints; checkpoint and sparsity factor tuned on DiaretDB1 itself
González-Gonzalo et al. [42]	Lesion localization	Attribution then inpainting, repeated until predicted normal	Saliency, guided backprop, IG, Grad-CAM, and Guided Grad-CAM (FROC, sensitivity at 10 FPs)	EyePACS, DiaretDB1, EUGENDA, and AREDS	One map for all abnormalities, not per lesion type (authors’ future work); up to 20 inpainting passes
Papadopoulos et al. [28]	Referable-DR detection + localization	Gated-attention pooling over 64 × 64 patches	Attention heatmaps (AUPR)	EyePACS, Messidor-2, and IDRiD	Attention not per lesion: attention AUC falls from 0.80 for any lesion to 0.75–0.76 for a specific type (three types)
EL-DETR [48]	Lesion detection (endoscopy/MRI/CT)	Decoder with separated content and position queries	Per-layer decoder cross-attention; Grad-CAM	Colonoscopy, MICCAI, KUMC (endoscopy); Brain Tumor (MRI/CT)	Needs bounding-box labels; attention shown, not scored
Lesion-Aware Transformer [21]	DR grading + lesion localization	Pixel-relation encoder; lesion filters with filter self-attention (K = 4), importance and diversity terms	Lesion activation maps (cross-attention)	Messidor-1, Messidor-2, and EyePACS	Four filters learned dataset-wide, not tied to named lesions; localization not scored
DeepDR [51]	Grading + lesion detection	Per-quadrant ResNet detector and Mask R-CNN feeding a grading head	Lesion masks overlaid	Private cohorts (SIM, CNDCS, and NDSP) and EyePACS	Lesion AUC per quadrant, not per image; splits not released
CLAT [52]	Disease diagnosis + lesion detection	One learnable token per lesion; cross-attention classifier	Concept weights; saliency maps	DDR-subset, FGADR, FGADDR, and private	Image-level labels from mask existence; AUC averaged over four lesions; 10-fold CV on a custom subset
Ours	Multi-label lesion detection	Per-lesion cross-attention, mask-supervised by a KL localization loss in training	Per-lesion attention maps (AIR, AUC-Judd, and deletion/insertion)	DDR, TJDR, IDRiD, and e-ophtha	Masks needed for part of training; MA weak on DDR

DR, diabetic retinopathy; AUC, area under the receiver operating characteristic (ROC) curve; AIR, Attention-Inside-Retina; CNN, convolutional neural network; GCN, graph convolutional network; MIL, multiple-instance learning; WSL, weakly supervised learning; IG, Integrated Gradients; FPs, false positives; CV, cross-validation; KL, Kullback–Leibler; AUPR, area under the precision–recall curve; MRI, magnetic resonance imaging; CT, computed tomography.

**Table 2:** Datasets, roles, and image counts.

Dataset	Role	# Images	Mask Format
DDR (grade data)	Pretrain grades (Stage 1)	11,767	ICDR grade labels (no masks)
APTOS 2019	Pretrain grades (Stage 1)	3662	ICDR grade labels (no masks)
DDR (mask data)	In-domain fine-tune + test (Stage 2)	757	Pixel-level lesion masks
TJDR	In-domain fine-tune + test (Stage 2)	561	Pixel-level lesion masks
IDRiD	Generalization (test only)	81	Pixel-level lesion masks
e-ophtha	Generalization (test only; MA + EX)	381 (MA)/82 (EX)	Pixel-level masks (MA, EX)

ICDR, International Clinical Diabetic Retinopathy.

**Table 3:** Per-lesion positive/negative counts (test sets).

Dataset	MA (pos/neg)	HE (pos/neg)	EX (pos/neg)	SE (pos/neg)
DDR	124/101	194/31	171/54	42/183
TJDR	37/76	69/44	67/46	41/72
IDRiD	81/0	80/1	81/0	40/41
e-ophtha	148/233	—	47/35	—

**Table 4:** Stage 1 metrics (validation).

QWK	Accuracy	Macro AUC	Macro AP
0.922	0.888	0.957	0.742

QWK, quadratic weighted kappa; AP, average precision.

**Table 5:** Stage 2 detection (test sets).

Dataset	Lesion	AUC	AUC 95% CI	AP	AP 95% CI	F1
*In domain*						
DDR	MA	0.584 ± 0.013	[0.504, 0.661]	0.609 ± 0.011	[0.525, 0.703]	0.714 ± 0.002
DDR	HE	0.938 ± 0.007	[0.901, 0.967]	0.990 ± 0.001	[0.982, 0.996]	0.948 ± 0.004
DDR	EX	0.926 ± 0.004	[0.886, 0.960]	0.974 ± 0.001	[0.956, 0.988]	0.926 ± 0.002
DDR	SE	0.814 ± 0.006	[0.740, 0.878]	0.595 ± 0.015	[0.441, 0.731]	0.469 ± 0.015
TJDR	MA	0.906 ± 0.003	[0.829, 0.967]	0.863 ± 0.007	[0.758, 0.940]	0.787 ± 0.011
TJDR	HE	0.925 ± 0.003	[0.872, 0.965]	0.958 ± 0.001	[0.922, 0.982]	0.841 ± 0.003
TJDR	EX	0.896 ± 0.013	[0.834, 0.950]	0.914 ± 0.010	[0.845, 0.967]	0.840 ± 0.011
TJDR	SE	0.832 ± 0.007	[0.755, 0.903]	0.777 ± 0.006	[0.661, 0.874]	0.615 ± 0.009
*Out of domain*						
IDRiD	MA	—	—	1.000 ± 0.000	—	0.998 ± 0.003
IDRiD	HE	—	—	1.000 ± 0.000	[0.998, 1.000]	0.976 ± 0.003
IDRiD	EX	—	—	1.000 ± 0.000	—	0.997 ± 0.004
IDRiD	SE	0.901 ± 0.005	[0.827, 0.959]	0.918 ± 0.004	[0.843, 0.966]	0.823 ± 0.031
e-ophtha	MA	0.962 ± 0.008	[0.944, 0.975]	0.940 ± 0.016	[0.911, 0.963]	0.788 ± 0.031
e-ophtha	EX	0.958 ± 0.003	[0.899, 0.996]	0.978 ± 0.002	[0.946, 0.997]	0.892 ± 0.031

Mean ± standard deviation (SD) across runs; 95% confidence interval (CI) in brackets. —: not computed (on IDRiD, MAs and EXs are in every image and HEs in all but one).

**Table 6:** Microaneurysm AUC against each group of MA-negative images.

MA-Negative Images	Dataset	MA-Positive/MA-Negative Images	AUC	95% CI
Healthy (no lesions)	e-ophtha	148/233	0.962 ± 0.008	[0.944, 0.975]
Healthy (no lesions)	TJDR	37/9	0.942 ± 0.006	[0.863, 0.998]
With exudates only	TJDR	37/29	0.949 ± 0.002	[0.889, 0.991]
With hemorrhages	TJDR	37/38	0.866 ± 0.005	[0.763, 0.944]
With hemorrhages	DDR	124/100	0.580 ± 0.013	[0.499, 0.656]

Means ± SDs across runs; 95% CI in brackets.

**Table 7:** Small hemorrhages in the MA-negative test images.

	DDR	TJDR
Small hemorrhages in images without microaneurysms	2322	40
Diameter (px): microaneurysms/small hemorrhages	5.0/4.5	6.2/5.9
Contrast: microaneurysms/small hemorrhages	0.045/0.044	0.126/0.099

Small hemorrhage: at most 60 pixels (90th percentile of microaneurysm size). Medians; contrast: how much darker the spot is than the retina around it.

**Table 8:** Attention localization on the DDR and TJDR test sets.

	DDR				TJDR			
Lesion	*n*	AUC-Judd	95% CI	Pixel AP	*n*	AUC-Judd	95% CI	Pixel AP
MA	124	0.952 ± 0.001	[0.938, 0.962]	0.015 ± 0.001	37	0.949 ± 0.002	[0.938, 0.976]	0.012 ± 0.001
HE	194	0.948 ± 0.001	[0.940, 0.959]	0.196 ± 0.002	69	0.992 ± 0.000	[0.987, 0.997]	0.235 ± 0.011
EX	171	0.979 ± 0.000	[0.975, 0.983]	0.123 ± 0.004	67	0.982 ± 0.001	[0.975, 0.989]	0.052 ± 0.008
SE	42	0.990 ± 0.000	[0.973, 0.994]	0.236 ± 0.017	41	0.996 ± 0.000	[0.993, 0.999]	0.314 ± 0.026

*n*: test images that contain the lesion. Each value is computed per image, and the median is reported; means ± SDs across runs; 95% CI in brackets.

**Table 9:** Deletion/insertion faithfulness.

Source	Lesion	*n*	Deletion	Insertion
DDR	MA	124	0.495 ± 0.005	0.848 ± 0.014
DDR	HE	194	0.540 ± 0.017	0.890 ± 0.008
DDR	EX	171	0.603 ± 0.018	0.859 ± 0.006
DDR	SE	42	0.459 ± 0.007	0.646 ± 0.011
TJDR	MA	37	0.483 ± 0.003	0.809 ± 0.013
TJDR	HE	69	0.532 ± 0.022	0.825 ± 0.013
TJDR	EX	67	0.625 ± 0.021	0.792 ± 0.012
TJDR	SE	41	0.468 ± 0.006	0.662 ± 0.006

*n*: test images that contain the lesion. Deletion and insertion: area under the lesion-probability curve as the most-attended pixels are removed or added back (Section 3.6.2); deletion: lower is better; insertion: higher is better. Means ± SDs across runs; 95% CIs: Table A1.

**Table 10:** Ablation of the localization loss (λ=0.3 vs. λ=0).

Source	Lesion	λ	AUC	AP	AIR	AUC-Judd
DDR	MA	0.3	**0.584 ± 0.013**	**0.609 ± 0.011**	**0.992 ± 0.001** *	**0.952 ± 0.001** *
		0	0.567 ± 0.007	0.575 ± 0.009	0.797 ± 0.022	0.777 ± 0.066
DDR	HE	0.3	0.938 ± 0.007	**0.990 ± 0.001**	**0.988 ± 0.001** *	**0.948 ± 0.001** *
		0	0.938 ± 0.004	0.989 ± 0.001	0.794 ± 0.021	0.780 ± 0.086
DDR	EX	0.3	**0.926 ± 0.004**	**0.974 ± 0.001**	**0.992 ± 0.000** *	**0.979 ± 0.000** *
		0	0.909 ± 0.013	0.964 ± 0.007	0.837 ± 0.021	0.933 ± 0.014
DDR	SE	0.3	**0.814 ± 0.006**	**0.595 ± 0.015**	**0.991 ± 0.001** *	**0.990 ± 0.000** *
		0	0.799 ± 0.026	0.587 ± 0.033	0.685 ± 0.082	0.873 ± 0.037
TJDR	MA	0.3	0.906 ± 0.003	0.863 ± 0.007	**0.988 ± 0.001** *	**0.949 ± 0.002** *
		0	0.906 ± 0.010	**0.866 ± 0.012**	0.802 ± 0.020	0.751 ± 0.141
TJDR	HE	0.3	**0.925 ± 0.003** *	**0.958 ± 0.001** *	**0.988 ± 0.001** *	**0.992 ± 0.000** *
		0	0.861 ± 0.012	0.926 ± 0.005	0.786 ± 0.013	0.815 ± 0.140
TJDR	EX	0.3	**0.896 ± 0.013**	**0.914 ± 0.010**	**0.992 ± 0.001** *	**0.982 ± 0.001** *
		0	0.859 ± 0.015	0.903 ± 0.010	0.790 ± 0.039	0.917 ± 0.029
TJDR	SE	0.3	**0.832 ± 0.007**	**0.777 ± 0.006**	**0.991 ± 0.000** *	**0.996 ± 0.000** *
		0	0.803 ± 0.018	0.726 ± 0.019	0.713 ± 0.050	0.800 ± 0.139

Means ± SDs across runs. Bold: better value; *: significant difference (Section 3.8); 95% CIs: Table A2.

**Table 11:** Deletion/insertion faithfulness with and without the localization loss.

Source	Lesion	*n*	λ	Deletion	Drop	Insertion	Gain
DDR	MA	124	0.3	**0.495 ± 0.005** *	**0.362 ± 0.007**	**0.848 ± 0.014**	**0.358 ± 0.010**
			0	0.610 ± 0.051	0.256 ± 0.039	0.833 ± 0.031	0.313 ± 0.049
DDR	HE	194	0.3	**0.540 ± 0.017** *	**0.370 ± 0.018**	0.890 ± 0.008	0.375 ± 0.042
			0	0.707 ± 0.055	0.211 ± 0.053	**0.908 ± 0.011**	**0.403 ± 0.024**
DDR	EX	171	0.3	**0.603 ± 0.018** *	**0.258 ± 0.020**	0.859 ± 0.006	**0.178 ± 0.008** *
			0	0.666 ± 0.021	0.184 ± 0.020	**0.878 ± 0.018**	0.098 ± 0.022
DDR	SE	42	0.3	**0.459 ± 0.007**	**0.233 ± 0.006**	**0.646 ± 0.011** *	**0.224 ± 0.030** *
			0	0.488 ± 0.042	0.207 ± 0.053	0.597 ± 0.011	0.121 ± 0.054
TJDR	MA	37	0.3	**0.483 ± 0.003**	**0.313 ± 0.006**	**0.809 ± 0.013**	**0.319 ± 0.009**
			0	0.531 ± 0.039	0.267 ± 0.033	0.794 ± 0.042	0.265 ± 0.052
TJDR	HE	69	0.3	**0.532 ± 0.022** *	**0.274 ± 0.021**	**0.825 ± 0.013**	**0.312 ± 0.039**
			0	0.649 ± 0.037	0.146 ± 0.033	0.819 ± 0.010	0.307 ± 0.036
TJDR	EX	67	0.3	**0.625 ± 0.021** *	**0.164 ± 0.023**	0.792 ± 0.012	**0.093 ± 0.008**
			0	0.734 ± 0.017	0.029 ± 0.014	**0.822 ± 0.011**	0.038 ± 0.034
TJDR	SE	41	0.3	**0.468 ± 0.006**	**0.260 ± 0.012**	**0.662 ± 0.006** *	**0.237 ± 0.024** *
			0	0.520 ± 0.039	0.181 ± 0.053	0.587 ± 0.009	0.133 ± 0.056

As in Table 9. Drop: probability on the original image minus deletion; gain: insertion minus probability on the blurred image (higher is better for both). Bold: better value; *: significant difference (Section 3.8); 95% CIs: Table A1.

**Table 12:** Where the first restored pixels fall during insertion.

				Lesion Area Restored		Outside the Retina	
Source	Lesion	*n*	*λ*	10%	20%	10%	20%
DDR	HE	194	0.3	**0.821 ± 0.004** *	**0.909 ± 0.003** *	**0.009 ± 0.000** *	**0.015 ± 0.001** *
			0	0.585 ± 0.102	0.642 ± 0.107	0.167 ± 0.088	0.212 ± 0.079
DDR	EX	171	0.3	**0.942 ± 0.001** *	**0.980 ± 0.000** *	**0.008 ± 0.000** *	**0.013 ± 0.000** *
			0	0.813 ± 0.022	0.867 ± 0.025	0.105 ± 0.080	0.178 ± 0.107
TJDR	HE	69	0.3	**0.892 ± 0.000** *	**0.918 ± 0.002** *	**0.013 ± 0.001** *	**0.022 ± 0.001** *
			0	0.555 ± 0.149	0.602 ± 0.159	0.236 ± 0.053	0.252 ± 0.047
TJDR	EX	67	0.3	**0.883 ± 0.009** *	**0.932 ± 0.005** *	**0.008 ± 0.000** *	**0.014 ± 0.001** *
			0	0.618 ± 0.076	0.708 ± 0.063	0.243 ± 0.201	0.261 ± 0.182

*n*: test images that contain the lesion. Pixels are added back to a blurred image in order of attention (Section 3.6.2). Lesion area restored: share of the lesion’s pixels among the first 10% or 20% of the image restored (higher is better); outside the retina: share of those pixels that lie outside the retina (lower is better). Means ± SDs across runs. Bold: better value; *: significant difference (Section 3.8).

**Table 13:** Sensitivity to λ.

		Test mAP		AUC-Judd		Pixel AP	
*λ*	Val mAP	DDR	TJDR	DDR	TJDR	DDR	TJDR
0	0.9097	0.777	0.855	0.827	0.830	0.036	0.014
0.05	0.9335	0.792	0.853	0.960	0.978	0.135	0.116
0.1	0.9309	0.792	0.859	0.964	0.980	0.140	0.149
0.3 (ours)	**0.9344**	0.787	0.879	0.967	0.980	0.143	0.159
0.5	0.9314	0.793	0.876	0.967	0.981	0.140	0.152
1.0	0.9342	0.795	0.880	0.969	0.981	0.141	0.152

Means over the four lesions. Bold: best validation mAP.

**Table 14:** Change in detection AUC when each training component is removed.

Dataset	Lesion	Without Stage 1	ASL → BCE	ASL → BCE + LS	Without MixUp	Without EMA
DDR	MA	−0.027	+0.011	+0.000	−0.005	−0.013 *
DDR	HE	−0.056 *	−0.007	−0.014	+0.007	−0.003
DDR	EX	+0.002	−0.010	−0.014	−0.001	−0.002
DDR	SE	−0.056 *	+0.005	−0.001	+0.031 *	−0.010
TJDR	MA	−0.031	+0.010	+0.010	+0.002	−0.006
TJDR	HE	−0.025	−0.030 *	−0.032 *	−0.008	+0.016 *
TJDR	EX	−0.024	−0.027	−0.030 *	−0.006	+0.001
TJDR	SE	+0.027	−0.017	−0.025	+0.017	−0.007
e-ophtha	MA	−0.028 *	+0.004	−0.008 *	−0.004	−0.006 *
e-ophtha	EX	−0.015	−0.002	+0.004	+0.010	−0.004

Each value: AUC without the component minus AUC with it; a negative value means that the AUC is lower without the component. BCE, binary cross-entropy; LS, label smoothing; EMA, exponential moving average. *: significant difference (Section 3.8); 95% CIs: Table A3.

**Table 15:** Detection AUC of our model and U-Net.

Dataset	Lesion	Ours	U-Net	Difference [95% CI]
DDR	MA	0.583 ± 0.015	**0.596 ± 0.008**	−0.013 [−0.071, 0.043]
DDR	HE	**0.937 ± 0.008**	0.896 ± 0.014	+0.041 [−0.011, 0.108]
DDR	EX	**0.925 ± 0.004**	0.869 ± 0.004	+0.056 [0.014, 0.099] *
DDR	SE	0.814 ± 0.007	**0.822 ± 0.011**	−0.008 [−0.056, 0.043]
TJDR	MA	**0.907 ± 0.004**	0.851 ± 0.009	+0.056 [0.002, 0.118] *
TJDR	HE	**0.926 ± 0.003**	0.884 ± 0.022	+0.042 [0.003, 0.089] *
TJDR	EX	**0.895 ± 0.015**	0.843 ± 0.022	+0.052 [−0.012, 0.124]
TJDR	SE	**0.831 ± 0.008**	0.782 ± 0.014	+0.049 [−0.026, 0.123]
IDRiD	SE	0.900 ± 0.005	**0.917 ± 0.004**	−0.017 [−0.063, 0.024]
e-ophtha	MA	**0.960 ± 0.009**	0.944 ± 0.001	+0.016 [0.002, 0.032] *
e-ophtha	EX	0.960 ± 0.001	**0.978 ± 0.007**	−0.018 [−0.065, 0.014]
Mean AUC		**0.876 ± 0.003**	0.853 ± 0.003	+0.023

Means ± SDs across runs. Difference: our AUC minus U-Net’s; a positive value means that our model is better; 95% CI in brackets. Bold: better value; *: significant difference (Section 3.8).

**Table 16:** Localization of our attention maps and U-Net probability maps.

Dataset	Lesion	AUC-Judd, Ours	AUC-Judd, U-Net	Pixel AP, Ours	Pixel AP, U-Net
DDR	MA	0.952 ± 0.001	**0.982 ± 0.004** *	0.016 ± 0.000	**0.230 ± 0.018**
DDR	HE	**0.949 ± 0.001** *	0.914 ± 0.013	0.195 ± 0.003	**0.506 ± 0.007**
DDR	EX	0.980 ± 0.000	**0.986 ± 0.003**	0.121 ± 0.003	**0.575 ± 0.008**
DDR	SE	**0.989 ± 0.000**	0.956 ± 0.017	0.239 ± 0.019	**0.511 ± 0.005**
TJDR	MA	0.949 ± 0.002	**0.986 ± 0.006**	0.012 ± 0.001	**0.281 ± 0.004**
TJDR	HE	**0.992 ± 0.000**	0.985 ± 0.007	0.240 ± 0.005	**0.666 ± 0.007**
TJDR	EX	0.982 ± 0.001	**0.983 ± 0.001**	0.050 ± 0.007	**0.477 ± 0.016**
TJDR	SE	**0.997 ± 0.000**	0.984 ± 0.010	0.325 ± 0.019	**0.654 ± 0.072**

Each value is computed per image, and the median is reported; means ± SDs across runs. Bold: better value; *: significant difference (Section 3.8).

**Table 17:** Our model trained with masks on half of the training images.

Dataset	Lesion	Detection AUC	AUC-Judd	Pixel AP
DDR	MA	0.598	0.945	0.014
DDR	HE	0.939	0.945	0.182
DDR	EX	0.915	0.977	0.120
DDR	SE	0.811	0.988	0.227
TJDR	MA	0.908	0.951	0.010
TJDR	HE	0.913	0.991	0.209
TJDR	EX	0.888	0.982	0.044
TJDR	SE	0.808	0.996	0.285

Masks on half of the training images and image-level labels on all of them (Section 3.7.2). AUC-Judd and pixel AP: median over images containing the lesion.

**Table 18:** Attention and detection on test images that lack a lesion but contain other lesions.

			Attention on Other Lesions (%)		AUC	
Source	Lesion Absent	*n*	*λ* = 0.3	*λ* = 0	*λ* = 0.3	*λ* = 0
DDR	MA	101	5.2 ± 0.1	**2.2 ± 0.2**	**0.584 ± 0.013**	0.567 ± 0.007
DDR	HE	31	2.2 ± 0.1	**0.4 ± 0.1**	0.938 ± 0.007	0.938 ± 0.004
DDR	EX	54	1.9 ± 0.1	**1.0 ± 0.1**	**0.926 ± 0.004**	0.909 ± 0.013
DDR	SE	183	5.9 ± 0.3	**2.3 ± 0.6**	**0.814 ± 0.006**	0.799 ± 0.026
TJDR	MA	67	2.5 ± 0.1	**0.3 ± 0.0**	0.902 ± 0.004	**0.903 ± 0.010**
TJDR	HE	35	1.6 ± 0.1	**0.2 ± 0.0**	**0.919 ± 0.004**	0.847 ± 0.020
TJDR	EX	37	2.2 ± 0.2	**0.6 ± 0.1**	**0.895 ± 0.012**	0.850 ± 0.014
TJDR	SE	63	3.8 ± 0.2	**0.8 ± 0.2**	**0.823 ± 0.006**	0.785 ± 0.014

*n*: test images without the lesion that contain other lesions. Attention on other lesions: share of the lesion’s attention map on the masks of the other lesions (lower is better). AUC: images with the lesion against these *n* images. Means ± SDs across runs. Bold: better value.

**Table 19:** Detection AUC on altered test images.

Dataset	Lesion	Original	Darker (×0.7)	Brighter (×1.3)	Lower Contrast (×0.7)	Blur (σ=2)	JPEG (Quality 30)	Sensor Noise (σ=8)
DDR	MA	0.589	0.622	0.584	0.608	0.600	0.601	0.520
DDR	HE	0.942	0.943	0.938	0.925	0.905	0.866	0.822
DDR	EX	0.928	0.923	0.924	0.912	0.835	0.831	0.770
DDR	SE	0.813	0.799	0.812	0.791	0.781	0.701	0.701
DDR	Mean	0.818	0.822	0.815	0.809	0.780	0.750	0.703
TJDR	MA	0.905	0.909	0.904	0.897	0.865	0.812	0.452
TJDR	HE	0.923	0.932	0.899	0.909	0.911	0.896	0.603
TJDR	EX	0.902	0.902	0.906	0.881	0.880	0.884	0.596
TJDR	SE	0.837	0.834	0.865	0.834	0.807	0.746	0.504
TJDR	Mean	0.892	0.894	0.893	0.880	0.866	0.834	0.539

One λ=0.3 model. Alterations are applied to the photograph before preprocessing; σ in gray levels.

## Data Availability

All datasets used in this study are publicly available and de-identified. DDR provides both image-level severity grades and pixel-level lesion annotations; TJDR, IDRiD, and e-ophtha provide pixel-level lesion annotations; and the APTOS 2019 set provides image-level severity grades. Each was used under its respective license, and no private or identifiable patient data were collected or generated.

## Abbreviations

The following abbreviations are used in this manuscript:

DR	Diabetic retinopathy
MA	Microaneurysm
HE	Hemorrhage
EX	Hard exudate
SE	Soft exudate
AIR	Attention-Inside-Retina
AUC	Area under the ROC curve
AUC-Judd	Area under the ROC curve, Judd variant
AP	Average precision
mAP	Mean average precision
FROC	Free-response receiver operating characteristic
ASL	Asymmetric loss
KL	Kullback–Leibler
CLAHE	Contrast-limited adaptive histogram equalization
ICDR	International Clinical Diabetic Retinopathy
QWK	Quadratic weighted kappa
CNN	Convolutional neural network
OCT	Optical coherence tomography
SD	Standard deviation
CI	Confidence interval
BCE	Binary cross-entropy
EMA	Exponential moving average

## Appendix A

Here, we present the 95% bootstrap confidence intervals of the run means over the test images (1000 resamples) for the comparisons with and without the localization loss; the means, standard deviations and significance marks are given in Table 10 and Table 11. For the training components, the 95% CIs of the AUC changes are given in Table A3.

**Table A1:** Deletion/insertion faithfulness: 95% CIs.

Source	Lesion	λ	Deletion	Drop	Insertion	Gain
DDR	MA	0.3	[0.491, 0.500]	[0.343, 0.379]	[0.833, 0.862]	[0.344, 0.371]
		0	[0.599, 0.622]	[0.239, 0.272]	[0.820, 0.846]	[0.299, 0.326]
DDR	HE	0.3	[0.535, 0.546]	[0.355, 0.386]	[0.877, 0.901]	[0.361, 0.387]
		0	[0.693, 0.721]	[0.195, 0.226]	[0.898, 0.918]	[0.393, 0.412]
DDR	EX	0.3	[0.598, 0.608]	[0.242, 0.274]	[0.846, 0.871]	[0.165, 0.191]
		0	[0.652, 0.679]	[0.167, 0.200]	[0.870, 0.886]	[0.090, 0.106]
DDR	SE	0.3	[0.449, 0.468]	[0.178, 0.288]	[0.597, 0.696]	[0.174, 0.274]
		0	[0.471, 0.507]	[0.171, 0.245]	[0.555, 0.633]	[0.079, 0.166]
TJDR	MA	0.3	[0.476, 0.489]	[0.260, 0.359]	[0.773, 0.841]	[0.283, 0.351]
		0	[0.507, 0.558]	[0.222, 0.312]	[0.761, 0.827]	[0.234, 0.297]
TJDR	HE	0.3	[0.525, 0.540]	[0.234, 0.311]	[0.794, 0.855]	[0.281, 0.344]
		0	[0.625, 0.674]	[0.111, 0.179]	[0.786, 0.852]	[0.274, 0.343]
TJDR	EX	0.3	[0.617, 0.632]	[0.142, 0.187]	[0.772, 0.813]	[0.073, 0.116]
		0	[0.721, 0.747]	[0.001, 0.058]	[0.806, 0.837]	[0.022, 0.054]
TJDR	SE	0.3	[0.458, 0.478]	[0.216, 0.301]	[0.623, 0.704]	[0.195, 0.275]
		0	[0.507, 0.534]	[0.146, 0.216]	[0.557, 0.616]	[0.103, 0.163]

These are the 95% bootstrap CIs of the run means over the test images; the means ± SDs are given in Table 9 and Table 11.

**Table A2:** Ablation of the localization loss: 95% CIs.

Source	Lesion	λ	AUC	AP	AIR	AUC-Judd
DDR	MA	0.3	[0.504, 0.661]	[0.525, 0.703]	[0.990, 0.993]	[0.938, 0.962]
		0	[0.490, 0.641]	[0.495, 0.667]	[0.793, 0.801]	[0.745, 0.824]
DDR	HE	0.3	[0.901, 0.967]	[0.982, 0.996]	[0.986, 0.989]	[0.940, 0.959]
		0	[0.899, 0.970]	[0.981, 0.996]	[0.791, 0.796]	[0.743, 0.812]
DDR	EX	0.3	[0.886, 0.960]	[0.956, 0.988]	[0.989, 0.994]	[0.975, 0.983]
		0	[0.858, 0.950]	[0.938, 0.984]	[0.833, 0.841]	[0.920, 0.945]
DDR	SE	0.3	[0.740, 0.878]	[0.441, 0.731]	[0.989, 0.993]	[0.973, 0.994]
		0	[0.724, 0.867]	[0.449, 0.733]	[0.660, 0.710]	[0.808, 0.926]
TJDR	MA	0.3	[0.829, 0.967]	[0.758, 0.940]	[0.984, 0.992]	[0.938, 0.976]
		0	[0.836, 0.962]	[0.769, 0.943]	[0.798, 0.807]	[0.646, 0.816]
TJDR	HE	0.3	[0.872, 0.965]	[0.922, 0.982]	[0.985, 0.991]	[0.987, 0.997]
		0	[0.793, 0.920]	[0.880, 0.960]	[0.782, 0.790]	[0.731, 0.882]
TJDR	EX	0.3	[0.834, 0.950]	[0.845, 0.967]	[0.989, 0.994]	[0.975, 0.989]
		0	[0.789, 0.919]	[0.838, 0.953]	[0.782, 0.798]	[0.863, 0.939]
TJDR	SE	0.3	[0.755, 0.903]	[0.661, 0.874]	[0.986, 0.994]	[0.993, 0.999]
		0	[0.724, 0.880]	[0.608, 0.842]	[0.678, 0.746]	[0.691, 0.917]

These are the 95% bootstrap CIs of the run means over the test images; the means ± SDs are given in Table 10.

**Table A3:** Change in detection AUC when each training component is removed: 95% CIs.

Dataset	Lesion	Without Stage 1	ASL → BCE	ASL → BCE + LS	Without MixUp	Without EMA
DDR	MA	[−0.075, 0.018]	[−0.007, 0.033]	[−0.020, 0.023]	[−0.018, 0.009]	[−0.026, −0.002]
DDR	HE	[−0.114, −0.012]	[−0.025, 0.009]	[−0.040, 0.007]	[−0.003, 0.019]	[−0.015, 0.008]
DDR	EX	[−0.023, 0.029]	[−0.026, 0.005]	[−0.035, 0.002]	[−0.012, 0.009]	[−0.012, 0.008]
DDR	SE	[−0.114, −0.002]	[−0.046, 0.053]	[−0.051, 0.044]	[0.0005, 0.068]	[−0.029, 0.007]
TJDR	MA	[−0.081, 0.019]	[−0.014, 0.032]	[−0.014, 0.034]	[−0.016, 0.018]	[−0.018, 0.005]
TJDR	HE	[−0.073, 0.026]	[−0.059, −0.003]	[−0.061, −0.006]	[−0.024, 0.008]	[0.0003, 0.037]
TJDR	EX	[−0.087, 0.030]	[−0.063, 0.002]	[−0.053, −0.008]	[−0.027, 0.013]	[−0.014, 0.016]
TJDR	SE	[−0.047, 0.105]	[−0.055, 0.019]	[−0.063, 0.015]	[−0.013, 0.051]	[−0.025, 0.013]
e-ophtha	MA	[−0.047, −0.012]	[−0.003, 0.011]	[−0.016, −0.0002]	[−0.009, 0.0001]	[−0.012, −0.001]
e-ophtha	EX	[−0.061, 0.034]	[−0.018, 0.013]	[−0.018, 0.028]	[−0.001, 0.032]	[−0.013, 0.003]

Paired bootstrap 95% CIs of the AUC without the component minus the AUC with it. When both bounds are below zero, the AUC is significantly lower without the component; when both are above zero, it is significantly higher; an interval that includes zero means no significant change. The changes themselves are given in Table 14.
